# LRRK2 and α-Synuclein: Distinct or Synergistic Players in Parkinson’s Disease?

**DOI:** 10.3389/fnins.2020.00577

**Published:** 2020-06-17

**Authors:** Darren M. O’Hara, Grishma Pawar, Suneil K. Kalia, Lorraine V. Kalia

**Affiliations:** ^1^Krembil Research Institute, Toronto Western Hospital, University Health Network, Toronto, ON, Canada; ^2^Department of Laboratory Medicine and Pathobiology, University of Toronto, Toronto, ON, Canada; ^3^Division of Neurosurgery, Department of Surgery, University of Toronto, Toronto, ON, Canada; ^4^Division of Neurology, Department of Medicine, University of Toronto, Toronto, ON, Canada; ^5^Edmond J. Safra Program in Parkinson’s Disease and the Morton and Gloria Shulman Movement Disorders Clinic, Division of Neurology, Department of Medicine, Toronto Western Hospital, University Health Network, Toronto, ON, Canada; ^6^Tanz Centre for Research in Neurodegenerative Diseases, University of Toronto, Toronto, ON, Canada

**Keywords:** Parkinson’s disease, neurodegeneration, α-synuclein, LRRK2, autophagy, mitochondria

## Abstract

Parkinson’s disease (PD) is the most common neurodegenerative movement disorder, characterized by prominent degeneration of dopaminergic neurons in the substantia nigra and aggregation of the protein α-synuclein within intraneuronal inclusions known as Lewy bodies. Ninety percent of PD cases are idiopathic while the remaining 10% are associated with gene mutations that affect cellular functions ranging from kinase activity to mitochondrial quality control, hinting at a multifactorial disease process. Mutations in *LRRK2* and *SNCA* (the gene coding for α-synuclein) cause monogenic forms of autosomal dominant PD, and polymorphisms in either gene are also associated with increased risk of idiopathic PD. Although Lewy bodies are a defining neuropathological feature of PD, an appreciable subset of patients with *LRRK2* mutations present with a clinical phenotype indistinguishable from idiopathic PD but lack Lewy pathology at autopsy, suggesting that *LRRK2*-mediated PD may occur independently of α-synuclein aggregation. Here, we examine whether LRRK2 and α-synuclein, as mediators of neurodegeneration in PD, exist in common or distinct pathways. Specifically, we review evidence from preclinical models and human neuropathological studies examining interactions between the two proteins. Elucidating the degree of interplay between LRRK2 and α-synuclein will be necessary for treatment stratification once effective targeted disease-modifying therapies are developed.

## Introduction

Parkinson’s disease (PD) is the most common neurodegenerative movement disorder, affecting 1% of people over the age of 65 (Kalia and Lang, 2015). It is characterized by the selective loss of dopaminergic neurons of the substantia nigra pars compacta (SN) resulting in progressive motor impairment. PD can also be associated with a variety of non-motor symptoms, including cognitive, psychiatric, sleep, and autonomic difficulties, and thus is a heterogenous disorder. An effective diagnostic test has yet to be identified. Currently, patients are deemed to have PD if they have met a number of clinical diagnostic criteria, but definitive diagnosis is not possible without post-mortem histopathological assessment. The main pathological hallmarks of PD are the loss of dopaminergic neurons in the SN and the accumulation of α-synuclein into large insoluble aggregates called Lewy bodies (LB), which are primarily composed of phosphorylated α-synuclein, p62, ubiquitin, and dysmorphic organelles and lipid membranes (Spillantini et al., 1997; Kalia and Kalia, 2015; Chartier and Duyckaerts, 2018; Shahmoradian et al., 2019). It is a matter of debate whether these LB are neuroprotective or neurotoxic, but a prevailing hypothesis within the field is that smaller aggregates of α-synuclein, particularly oligomers and small fibrils, are the neurotoxic forms (Danzer et al., 2007; Karpinar et al., 2009; Winner et al., 2011; Kalia et al., 2013; Bengoa-Vergniory et al., 2017) and it has been shown that these forms are present at degenerating sites in the diseased brain (Sharon et al., 2003; Tofaris et al., 2003; Periquet et al., 2007).

Mutations in *SNCA*, the gene coding for α-synuclein, and *LRRK2* are responsible for familial autosomal dominant PD (Polymeropoulos et al., 1997; Wszolek et al., 2004; Zimprich et al., 2004; Khan et al., 2005; Singleton, 2005). Studies have shown that the LRRK2 protein is present in LB, suggesting that LRRK2 and α-synuclein might interact with each other during the course of PD (Alegre-Abarrategui et al., 2008; Perry et al., 2008). However, PD patients with *LRRK2* mutations do not always have typical PD pathology at autopsy. It is now well established that there is a subset of *LRRK2*-associated PD patients who do not display Lewy pathology but may have aggregates of other proteins, such as tau and TDP-43 (Zimprich et al., 2004; Ling et al., 2013; Kalia et al., 2015; Henderson et al., 2019b), suggesting that PD due to LRRK2 dysfunction may occur independently of α-synuclein aggregation.

**FIGURE 1:**
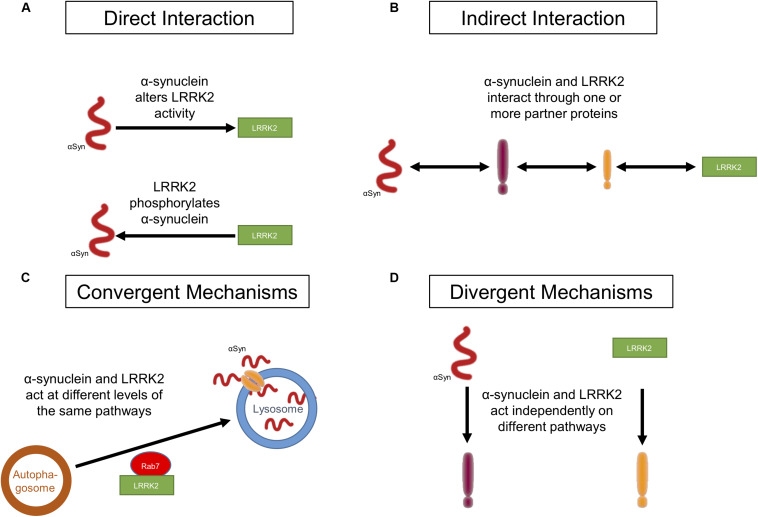
Potential ways in which LRRK2 and α-synuclein act in PD. **(A)** LRRK2 and α-synuclein affect each other through a direct physical interaction. **(B)** LRRK2 and α-synuclein affect each other through an indirect physical interaction in which a mediator(s), such as a molecular chaperone(s), links the two proteins. **(C)** Both LRRK2 and α-synuclein act synergistically on targets within the same molecular pathway without interacting with each other. **(D)** LRRK2 and α-synuclein do not interact at all and each affect targets in distinct molecular pathways.

In this review, we will examine the evidence from protein biochemistry, preclinical models, and human neuropathological studies for interactions between LRRK2 and α-synuclein (Figure 1) and discuss the role of these potential mechanisms in disease pathogenesis.

## Direct Interaction of LRRK2 and α-Synuclein

LRRK2 is a large protein that belongs to the ROCO protein superfamily. It is a complex multi-domain protein with a Ras of complex (ROC) GTPase domain, *C*-terminal of ROC (COR) linker region, and serine/threonine kinase domain. In addition, the protein contains a *N*-terminal ankyrin domain, a leucine-rich repeat (LRR), and a *C*-terminal WD40 domain (Bosgraaf and Van Haastert, 2003; Guaitoli et al., 2016), which all serve as protein–protein interaction domains. At least eight pathogenic LRRK2 mutations (G2019S, R1441G/H/C, I2012T, Y1699C, I2020T, and N1437H) are associated with autosomal dominant PD (Paisan-Ruiz et al., 2004; Zimprich et al., 2004; Chen and Wu, 2018). Among these mutations, G2019S is the most prevalent (Kachergus et al., 2005; Cresto et al., 2019) and occurs in the kinase domain, resulting in an increase in the kinase activity of LRRK2 (West et al., 2007; Cookson, 2012; Chen and Wu, 2018). LRRK2 is capable of undergoing autophosphorylation and this property of LRRK2 has been used as a readout of its kinase activity (Greggio, 2012; Sheng et al., 2012). The LRRK2 kinase domain contains an activation P-loop with a DFG (conserved residues Asp–Phe–Gly)-APE motif which controls the kinase activity. The glycine residue in the motif is highly conserved and its small side chain makes the activation loop flexible. The G2019S mutation changes the highly conserved glycine in the DFG motif to serine (Kachergus et al., 2005; Greggio and Cookson, 2009). It is speculated that the serine substitution makes the activation loop less flexible, thus locking the kinase domain of LRRK2 in an active conformation (Greggio and Cookson, 2009). Increased kinase activity of mutated LRRK2 is associated with enhanced cell death *in vitro* (Greggio et al., 2006; Smith et al., 2006; West et al., 2007) and deletion of the kinase domain or reduced kinase activity *in vitro* and *in vivo* can ameliorate the toxic effects of LRRK2 (Greggio et al., 2006; Smith et al., 2006; Volpicelli-Daley et al., 2016). In addition, LRRK2 kinase activity is required for the pathogenic effects of the G2019S LRRK2 mutation in rats (Cookson et al., 2007; Tsika et al., 2015).

A direct protein–protein interaction between LRRK2 and α-synuclein would involve the physical contact of the two proteins, allowing one to directly regulate the function and/or activity of the other (Figure 1A). Under normal physiological conditions, α-synuclein is present as a monomer in the cytosol of neurons or is associated with various membranes and vesicular structures (Jakes et al., 1994; Kahle et al., 2000; Pineda and Burré, 2017; Meade et al., 2019). However, under certain stress conditions, or due to other unknown causes, α-synuclein self-aggregates into oligomers and later into fibrils that form LB (Conway et al., 1998; Meade et al., 2019). Approximately 90% of α-synuclein deposited in LB in PD patients is phosphorylated at S129 (Fujiwara et al., 2002; Anderson et al., 2006; Walker et al., 2013). Since LRRK2 is a serine-threonine kinase, it has been suggested that mutant G2019S LRRK2 can directly interact with and phosphorylate α-synuclein, resulting in α-synuclein aggregation which eventually leads to cell death (Qing et al., 2009; Guerreiro et al., 2013). LRRK2 was found to co-localize with phosphorylated α-synuclein in human PD brain samples (Guerreiro et al., 2013). However, only one study to date has demonstrated direct phosphorylation of α-synuclein by LRRK2 at S129 (Qing et al., 2009). There is little other evidence to support direct phosphorylation of α-synuclein by LRRK2. Indeed, some studies have shown that phosphorylated α-synuclein levels are decreased or unchanged in mutant LRRK2 expressing mice, demonstrating that α-synuclein is not a substrate for LRRK2 kinase activity *in vivo* (Lin et al., 2009; Dusonchet et al., 2011). Furthermore, kinase deletion in LRRK2 accelerated the pathological features in mutant A53T α-synuclein/LRRK2 kinase deletion double transgenic mice when compared to A53T α-synuclein/wild-type (WT) LRRK2 mice, suggesting that the kinase activity does not promote mutant A53T α-synuclein-mediated neuropathology (Lin et al., 2009). Several other kinases, such as G-protein coupled receptor kinases, casein kinases, and polo-like kinases, have been implicated in phosphorylating α-synuclein (Okochi et al., 2000; Chen and Feany, 2005; Waxman and Giasson, 2011; Braithwaite et al., 2012; Tenreiro et al., 2014). Taken together, there is limited evidence to support a direct physical interaction between LRRK2 and α-synuclein or direct phosphorylation of α-synuclein by LRRK2. To the best of our knowledge, there is no evidence to date that α-synuclein can directly modulate LRRK2 activity. In the following section, we will discuss in detail the potential indirect interactions that may mediate LRRK2-dependent α-synuclein aggregation and neurodegeneration in PD.

## Indirect Interaction of LRRK2 and α-Synuclein

An indirect interaction between LRRK2 and α-synuclein, whereby they are common proteins in a larger complex, is a more likely scenario (Figure 1B). LRRK2 and α-synuclein have been co-immunoprecipitated from brain tissue extracts of human PD and dementia with Lewy body (DLB) patients, but not from age-matched control brains (Qing et al., 2009; Guerreiro et al., 2013). Both proteins have also been co-immunoprecipitated from transfected HEK293 cells under oxidative stress (Guerreiro et al., 2013). Studies have investigated the effect of overexpressing LRRK2 mutants on α-synuclein levels and aggregation in cell and transgenic animal models to determine if they do interact with each other. Increased kinase activity of mutant G2019S LRRK2 can induce a kinase-dependent increase in levels of phosphorylated α-synuclein, leading to its aggregation, and kinase inhibitors can prevent phosphorylated α-synuclein from forming protein inclusions (Volpicelli-Daley et al., 2016; Longo et al., 2017; Xiong et al., 2017). Transgenic mice and primary neurons expressing mutant G2019S LRRK2 showed an increase in neurodegeneration, somatic accumulation of α-synuclein, and aggregation in response to α-synuclein fibril exposure. These effects were not observed in mice and primary neurons expressing WT LRRK2 (Volpicelli-Daley et al., 2016; Bieri et al., 2019). These findings were replicated in human induced pluripotent stem cell (iPSC)-derived neurons from G2019S *LRRK2* carriers. These iPSC-derived neurons showed enhanced α-synuclein aggregation in response to exposure to α-synuclein fibrils (Bieri et al., 2019). In A53T α-synuclein/G2019S LRRK2 double transgenic mice, G2019S LRRK2 expression exacerbated A53T α-synuclein-mediated neurodegeneration and abnormal aggregation. WT LRRK2 did not seem to have any effect on the progression of A53T α-synuclein-mediated pathology in these double transgenic animal models (Lin et al., 2009). Similarly, co-transfection of SH-SY5Y cells with mutant G2019S LRRK2 and α-synuclein resulted in cytotoxicity and showed an increase in protein inclusions as compared to cells transfected with α-synuclein alone (WT or mutant A53T) (Kondo et al., 2011). These studies point towards an association between G2019S LRRK2 expression and α-synuclein pathology. Therefore, it is not surprising that LRRK2 inhibition can ameliorate these pathological features *in vitro* and *in vivo* (Lin et al., 2009; Guerreiro et al., 2013; Daher et al., 2014, 2015; Volpicelli-Daley et al., 2016; Xiong et al., 2017). These studies clearly identify a role for LRRK2 in α-synuclein-mediated cytotoxicity. They also provide evidence for an interaction between LRRK2 and α-synuclein, but this interaction is likely indirect. Although the exact mechanism of the interaction remains to be elucidated, current evidence points toward molecular chaperones as potential intermediary proteins.

### Chaperones

Molecular chaperones are a class of proteins that assist in protein folding and assembly of protein complexes, as well as in directing misfolded proteins to degradation pathways. Their central role in protein homeostasis, or proteostasis, makes their involvement in PD and other protein aggregation disorders an important area of research (Friesen et al., 2017). In recent years, the role of a subfamily called 14-3-3 proteins and their interactions with both α-synuclein and LRRK2 have been explored. 14-3-3 proteins represent 1% of total brain protein and have roles in a wide variety of neuronal functions, including control over cell death pathways (Dougherty and Morrison, 2004). 14-3-3 proteins share structural homology with α-synuclein and can also become sequestered in LB (Ostrerova et al., 1999; Kawamoto et al., 2002). 14-3-3 proteins are strong interactors with phosphorylated α-synuclein, which may explain why they are sequestered in LB where they can no longer exert an anti-apoptotic effect (McFarland et al., 2008). Expression of human 14-3-3θ or the *Caenorhabditis elegans* homolog, ftt-2, was capable of protecting dopaminergic neurons from α-synuclein-mediated toxicity in a transgenic *C. elegans* model (Yacoubian et al., 2010). One transgenic mouse model overexpressing α-synuclein showed a reduction in expression of 14-3-3θ, γ, and ε (Yacoubian et al., 2010). Another study showed that 14-3-3θ promotes the extracellular release of α-synuclein, but the released α-synuclein is less toxic and shows reduced oligomerization, seeding capability, and internalization. Conversely, 14-3-3 inhibition reduces the amount of α-synuclein released, yet the released α-synuclein is more toxic (Wang et al., 2018).

Interactions between LRRK2 and 14-3-3 proteins have also been well studied. It has been reported that LRRK2 binds to different isoforms of the 14-3-3 family upon auto-phosphorylation of LRRK2 at residues S910 and S935 (Dzamko et al., 2010). Thus, LRRK2 kinase activity may directly modulate binding of 14-3-3 proteins to LRRK2. Indeed, several of the common LRRK2 mutations show decreased phosphorylation at S910 and S935 in cell lines which is associated with disruption of the interaction between the two proteins (Nichols et al., 2010). PAK6 can phosphorylate 14-3-3γ at its S59 residue, which can promote dissociation from LRRK2 (Civiero et al., 2017). Disruption of the LRRK2-14-3-3 interaction alters LRRK2 localization within the cell (Mamais et al., 2014), whereas 14-3-3 binding to LRRK2 prevents dephosphorylation of LRRK2, stabilizing it in its active state (Civiero et al., 2017). Both LRRK2 and α-synuclein have been found in complexes with 14-3-3 proteins (Xu et al., 2002; Dzamko et al., 2010), and thus it is an attractive hypothesis that 14-3-3 proteins may act as an intermediary in an indirect interaction between LRRK2 and α-synuclein but further investigation is required before one can draw this conclusion.

The heat shock proteins, Hsp70 and Hsp90, are additional molecular chaperones that interact with both α-synuclein and LRRK2. Both of these chaperones have been identified as components of LB (McLean et al., 2002; Leverenz et al., 2007). HSP70 expression has been shown to prevent dopaminergic cell death in a *Drosophila* model of α-synuclein toxicity (Auluck et al., 2002; Fan et al., 2006), and overexpression of rat HSP70 reduces α-synuclein aggregation and toxicity in a mouse model overexpressing human α-synuclein (Klucken et al., 2004). These effects may be mediated by another chaperone called *C*-terminus of HSP70 interacting protein (CHIP). CHIP contains an *N*-terminal tetratricopeptide (TPR) domain which mediates its interaction with HSP70 and HSP90, as well as a U-box domain which confers E3 ligase activity (Ballinger et al., 1999). CHIP, HSP70, and α-synuclein form a complex in cultured cells, and overexpression of CHIP increases clearance of α-synuclein from cells, while knockdown of CHIP results in increases in the cellular load of oligomeric α-synuclein (Shin et al., 2005; Kalia et al., 2011). CHIP overexpression *in vivo* also appears to have protective effects as it reduces α-synuclein aggregation in rat brain (Dimant et al., 2014). CHIP can interact with LRRK2 in a TPR domain-dependent manner and ubiquitination of LRRK2 by CHIP *in vitro* causes proteasomal degradation of LRRK2 (Ko et al., 2009; Rudenko et al., 2017). The CHIP-LRRK2 interaction is mediated by HSP70 and/or HSP90 and, in this complex, HSP90 can interact with LRRK2 to mitigate CHIP-mediated degradation of LRRK2 (Ding and Goldberg, 2009; Ko et al., 2009). These results show that increasing the E3 ligase activity of CHIP and blocking HSP90 chaperone activity can increase LRRK2 degradation and mediate the toxic effects of overactive LRRK2. Interestingly, increased degradation of LRRK2 may be associated with an increased risk of disease. The G2385R LRRK2 mutation is a risk factor for PD and displays an increased affinity for CHIP, resulting in an increase in proteasomal degradation of LRRK2 (Rudenko et al., 2017). These data highlight the fine equilibrium that exists in maintaining LRRK2 activity above a certain minimum, but below a maximum threshold to prevent pathogenic activity.

Chaperones provide an intriguing link between LRRK2, α-synuclein, and protein degradation. However, evidence that they may act as intermediaries facilitating an indirect interaction between the proteins is limited. The Bcl-2 associated athanogene (BAG) family of proteins are known to regulate the activity of heat shock proteins and 14-3-3 proteins, and have been implicated in PD pathogenesis (Ebrahimi-Fakhari et al., 2011; Kalia et al., 2011; Friesen et al., 2017). BAG5 has been nominated as a LRRK2 interactor, and it has been shown that BAG proteins are involved in the stabilization of LRRK2 binding pairs (Zheng et al., 2008; Xu et al., 2013; Beilina et al., 2014) and α-synuclein interactions with BAG proteins are well categorized (Kalia et al., 2011; Cao et al., 2017). Thus, molecular chaperones may act together to modulate both LRRK2 and α-synuclein under certain conditions. It is conceivable that the chaperone system may act as an intermediate, facilitating the degradation of both proteins under certain disease circumstances. Molecular chaperones may mediate interactions between LRRK2 and α-synuclein to promote their degradation under conditions where both proteins are dysfunctional, and defects in this system could lead to decreases in degradation and accumulation of α-synuclein or increased kinase activity of LRRK2. Further investigation is required to confirm these possibilities, but it is perhaps more likely that the two proteins converge on common pathways, such as protein degradation (Figure 1C). We will examine more well-defined common cellular pathways which may impact LRRK2-mediated accumulation of α-synuclein below (see section “Convergent Mechanisms”).

### Cell-to-Cell Transmission of α-Synuclein

Previously, α-synuclein was considered as a cell autonomous protein in that its cytotoxicity was thought to be restricted to the cell within which it aggregates. However, accumulating evidence suggests that α-synuclein from an affected cell can be released into the extracellular space and taken up by recipient cells, including neighboring neurons and glia (Volpicelli-Daley et al., 2011; Lee et al., 2012; Reyes et al., 2015; Lim et al., 2018). When small aggregates are taken up by recipient neurons, they can act as seeds for α-synuclein monomers leading to their aggregation and subsequent formation of protein inclusions in recipient neurons (Lee et al., 2010; Hansen et al., 2011; Kordower et al., 2011; Angot et al., 2012). Two studies showed that α-synuclein aggregates, similar to LB, were found in grafted neurons of PD patients transplanted with fetal mesencephalic dopaminergic neurons (Kordower et al., 2008; Li et al., 2008). Neuron-to-neuron transmission of α-synuclein could be one mechanism that explains these findings. Spread of α-synuclein could also possibly explain the Braak hypothesis that Lewy pathology in PD patients undergoes a predictable distribution pattern starting in the lower brainstem and olfactory bulbs in prodromal disease, progressing to the midbrain region at disease diagnosis, and eventually reaching cortical regions at later disease stages (Braak et al., 2003). A transmission of α-synuclein from diseased to non-diseased neurons could be the underlying mechanism of PD progression.

**FIGURE 2:**
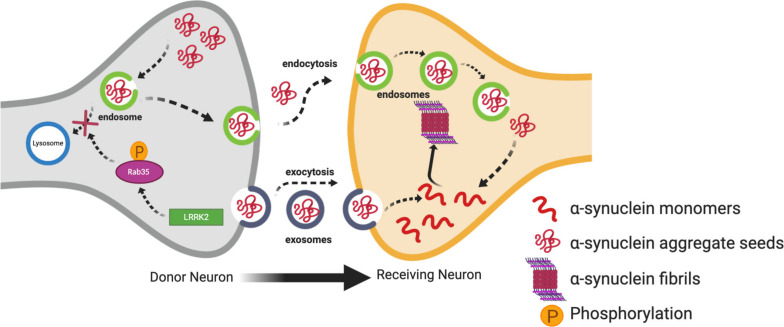
Contribution of mutant LRRK2 to cell-to-cell transmission of α-synuclein. Increased kinase activity of mutant LRRK2 can phosphorylate Rab35 affecting its interaction with its substrates, and eventually preventing the endosome-lysosmal degradation of α-synuclein aggregates. This in turn can increase the mobile cytosolic pools of α-synuclein aggregate seeds which can get released into the extracellular space in exosomes. These seeds can then be taken up into the cytosol of neighboring neurons where they can promote aggregation of α-synuclein monomers into oligomers and fibrils.

Studies have demonstrated that LRRK2 can regulate cell-to-cell transmission of α-synuclein (Kondo et al., 2011; Volpicelli-Daley et al., 2016). In support of this, one interesting study demonstrated the transmission of vesicles containing α-synuclein to neighboring neurons through conditioned media. This transmission of vesicles containing α-synuclein was enhanced in SH-SY5Y cells transfected with G2019S LRRK2 as compared to WT LRRK2 or α-synuclein alone. Levels of α-synuclein were also increased in conditioned media of cells transfected with G2019S LRRK2 as compared to controls (Kondo et al., 2011). Rab proteins provide an interesting link between LRRK2 and the propagation of α-synuclein. Rabs are a family of G proteins which are members of the Ras superfamily of proteins. They are commonly accepted as the main substrates of LRRK2 (Steger et al., 2016, 2017; Jeong et al., 2018; Pfeffer, 2018; Seol et al., 2019) and are known as the gatekeepers of membrane trafficking within the cell, regulating vesicle formation, movement along actin and tubulin cytoskeletons, and docking and fusion with other vesicles and organelles (Hutagalung and Novick, 2011; Bhuin and Roy, 2014; Zhen and Stenmark, 2015). Increased phosphorylation of Rabs by overexpressed LRRK2 mutants disturbs the interaction of Rabs with their substrates (Steger et al., 2016; Jeong et al., 2018). Dysregulation of Rabs can alter the endosome-lysosomal (E-L) pathway which degrades α-synuclein aggregates after being taken up by recipient neurons (Bourdenx et al., 2014). Therefore, it is tempting to speculate that this would in turn divert the α-synuclein aggregates to exocytosis leading to cell-to-cell transmission (Figure 2). Rab35 plays an important role in regulating endosomal trafficking and recycling (Donaldson et al., 2016; Song and Testa, 2018). A recent study demonstrated that phosphorylation of Rab35 by mutant LRRK2 is essential for LRRK2-stimulated α-synuclein propagation (Bae et al., 2018). Specifically, phospho-null Rab35 was shown to reduce α-synuclein propagation in *C. elegans*, and α-synuclein and Rab35-positive endosomes were found to co-localize in α-synuclein transgenic mice (Bae et al., 2018). Using a phosphomimetic mutant of Rab35, phosphorylation of Rab35 was found to be associated with neurotoxicity in primary cortical neurons. Similarly, AAV-mediated expression of the Rab35 phosphomimetic in the SN of rats resulted in substantially increased degeneration of dopaminergic neurons (Jeong et al., 2018). Increased Rab 35 expression *in vitro* has been shown to increase the aggregation of A53T α-synuclein (Chiu et al., 2016). Increased Rab35 levels have been found in PD mouse models, including the G2019S LRRK2 transgenic model, as well as in human PD post-mortem brains compared to age-matched controls (Chiu et al., 2016). Collectively, these studies suggest Rab35 is a potential link between LRRK2 mutants and α-synuclein propagation, lending weight to the hypothesis that dysfunction in the E-L system due to Rab phosphorylation by LRRK2 mutants may be important for the cell-to-cell transmission of α-synuclein. Dysregulation of another Rab protein, Rab7L1 (Rab29), by mutant LRRK2 has also been linked to PD through dysregulation of E-L trafficking (Simón-Sánchez et al., 2009; MacLeod et al., 2013; Beilina et al., 2014; Kuwahara et al., 2016; Tang, 2017; Liu et al., 2018). The *Rab7L1* gene has been a nominated as a candidate gene within the chromosome 1 locus identified by genome-wide association study (GWAS) for increased risk of PD (Simón-Sánchez et al., 2009). It is not clear yet if this interaction between LRRK2 and Rab7LI could eventually result in α-synuclein aggregation and transmission, but it will be discussed further in the context of autophagy below.

## Convergent Mechanisms

### Autophagy-Lysosomal Pathway

**FIGURE 3:**
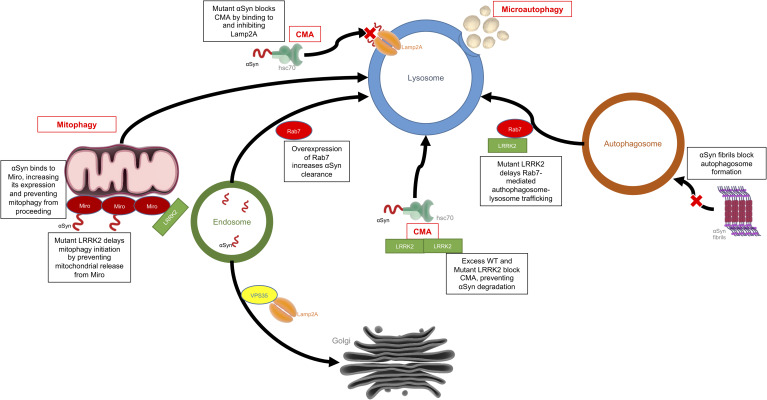
Effects of LRRK2 and α-synuclein on the autophagy-lysosomal pathway. This is an example of how LRRK2 and α-synuclein can have convergent mechanisms with both affecting the autophagy-lysosomal system but each acting on different targets within the system. Mutant or aggregated α-synuclein can block CMA and autophagosome formation, while mutant LRRK2 can also block CMA, disrupt mitophagy or delay autophagosome trafficking.

Autophagy is the process by which a cell can remove unnecessary or dysfunctional components, including misfolded proteins and damaged organelles via the lysosome. There are three main types of autophagy: macroautophagy, microautophagy, and chaperone-mediated autophagy (CMA). Macroautophagy involves the formation of double membraned vesicles, called autophagosomes, around portions of the cytoplasm which later fuse with lysosomes resulting in the degradation of their contents (Ravikumar et al., 2009). Microautophagy is mediated directly by the lysosome, which engulfs small portions of cytosolic components in a process of membrane invagination (Müller et al., 2000). As the name suggests, CMA is dependent on chaperones, such as heat shock cognate 70 (Hsc70), which bind target proteins containing a KFERQ motif and directly shuttle them across the lysosomal membrane by interaction with the lysosomal receptor, LAMP2a, excluding the requirement for the formation of additional vesicles (Massey et al., 2004). Defects in the autophagy systems have been observed in PD patient brains, and levels of LAMP2a and Hsc70 have been shown to be decreased within the SN (Chu et al., 2009; Alvarez-Erviti et al., 2010; Dehay et al., 2010). There is substantial evidence to suggest that the dysfunctional autophagy seen in patient brains could be due to accumulation of α-synuclein and/or an increase in the kinase activity of LRRK2, as discussed below (Figure 3).

#### α-Synuclein

α-Synuclein contains a CMA recognition motif and is partially degraded in a CMA-dependent manner in isolated liver lysosomes, cell lines, neurons, and *in vivo* (Webb et al., 2003; Cuervo et al., 2004; Lee et al., 2004; Mak et al., 2010). Mutant and aggregated forms of α-synuclein cannot be efficiently degraded by the lysosome, as they bind to LAMP2a blocking CMA (Cuervo et al., 2004; Lee et al., 2004). This effect has been demonstrated in primary neurons (Vogiatzi et al., 2008). Interestingly, dopamine modified α-synuclein further inhibits CMA, which may help explain the selective vulnerability of dopaminergic neurons in PD (Martinez-Vicente et al., 2008). Blocking expression of LAMP2a results in compensatory upregulation of macroautophagy, but α-synuclein accumulation has also been shown to negatively affect this process by blockage of autophagosome formation (Massey et al., 2006; Winslow et al., 2010). VPS35 is another protein in which mutations are associated with PD, and it has been shown to be critical for retrieval of LAMP2a from the endosome to the Golgi, and defects in this system can result in α-synuclein aggregation in dopaminergic neurons (Tang et al., 2015). Enhancement of autophagy has also been shown to induce clearance of aggregated α-synuclein *in vitro* and *in vivo*, supporting the notion that targeting this pathway may be a therapeutic approach for disease modification in PD (Moors et al., 2017; Suresh et al., 2017, 2018).

#### LRRK2

LRRK2 also contains several CMA targeting motifs, and it has been shown that knockdown of LAMP2a is associated with reduction of LRRK2 degradation to ∼50% of normal levels, resulting in increased intracellular levels of LRRK2. Conversely, mutant LRRK2 can block CMA by inhibiting LAMP2a (Orenstein et al., 2013). LRRK2 has been shown to regulate lysosomal protein trafficking and morphology, and fibroblasts from PD patients with *LRRK2*-related PD mutations show altered lysosomal morphology due to the increased influx of Ca^2+^ into the lysosome (Marchant and Patel, 2015). This effect can be reversed by inhibiting the two-pore calcium channels present on all acidic vesicles (endosomes and lysosomes) of the E-L system (Hockey et al., 2015; Kuwahara et al., 2016; Eguchi et al., 2018). LRRK2 knockout (KO) mice show defective protein clearance and increased accumulation of α-synuclein, resulting in increases in apoptotic cell death and oxidative damage (Tong et al., 2010). Overexpression of G2019S LRRK2 has also been associated with reduced degradation capacity and enlarged lysosomes in astrocytes, an effect which is directly related to the kinase activity of LRRK2 (Henry et al., 2015). Primary neurons cultured from G2019S LRRK2 knock-in mice showed significant changes in lysosomal morphology and acidification, resulting in decreased autophagic flux and a subsequent accumulation of insoluble α-synuclein; these effects could be reversed by inhibition of LRRK2 kinase activity (Schapansky et al., 2018). Other studies have shown that LRRK2 KO induces autophagy which suggests that the interaction of LRRK2 with the autophagy machinery is nuanced. As mentioned above, the best studied link between LRRK2 and the E-L system with respect to a role in PD is the phosphorylation of Rab proteins. Fourteen Rab family members have been identified to be phosphorylated by LRRK2 in their switch-II domains (Steger et al., 2016, 2017). Some of these Rabs, including Rab3a and Rab8a, promote vesicular trafficking from the ER to the Golgi apparatus which has been shown to reduce cytotoxicity associated with accumulation of α-synuclein in PD models (Gitler et al., 2008).

One of the most interesting connections between LRRK2 and the Rab proteins of the E-L system is Rab7. Rab7 plays a central role in the maturation of the autophagosome, as well as early-to-late endosomes (Jager et al., 2004). Mutant LRRK2 causes a delay in early-to-late and late endosomal trafficking, and fibroblasts from PD patients with pathogenic *LRRK2* mutations show decreased Rab7 activity compared to healthy controls (Gomez-Suaga et al., 2014). Rab7 also provides a link between LRRK2 and α-synuclein, as Rab7 increased the clearance of α-synuclein aggregates, reduced cell death, and rescued locomotor deficits in α-synuclein transgenic *Drosophila* (Dinter et al., 2016). Nuclear factor erythroid-2 related factor (Nrf2) is an antioxidant gene thought to be a potential therapeutic target for neurodegeneration (Shih et al., 2003). Activation of Nrf2 reduces toxicity associated with LRRK2 and α-synuclein by enhancing the accumulation of LRRK2 in inclusion bodies, and subsequently decreasing its activity in other parts of the neuron (Skibinski et al., 2017). These data highlight the possibility that mutant LRRK2 acts as a negative regulator of autophagy, and this leads to an accumulation of α-synuclein in dopaminergic neurons. In a feedback loop, α-synuclein itself disrupts the normal function of the E-L system and further α-synuclein accumulation occurs.

#### Glucocerebrosidase

Lysosomes are the endpoint of the autophagy-lysosomal pathway and serve as the main degradative component of the cell. Lysosomes have a low pH (4.5–5.5) which is established by the vacuolar H^+^-ATPase. This acidic environment provides optimal conditions for the action of degradative hydrolases which break down a vast array of macromolecules into base components of amino acids, fatty acids, and monosaccharides for export back into the cytosol for recycling (Perera and Zoncu, 2016). Defects in lysosomal function result in lysosomal storage disorders, a number of which are characterized by progressive neurodegeneration (Osellame and Duchen, 2014). One of the most common lysosomal storage disorders is Gaucher disease, and this results from the homozygous loss of function of the lysosomal enzyme β-glucocerebrosidase (GCase), and the subsequent accumulation of glucosylceramide (Brady et al., 1965; Stirnemann et al., 2017). Interestingly, Gaucher patients as well as carriers of heterozygous mutations in *GBA*, the gene that encodes GCase, are at increased risk of developing PD (Tayebi et al., 2001; Goker-Alpan et al., 2004; Sidransky et al., 2009; Platt, 2014; Gan-Or et al., 2015). There is accumulating evidence that build-up of glycosphingolipids due to dysfunction of GCase can also increase α-synuclein accumulation (Taguchi et al., 2017; Kim et al., 2018). Indeed, accumulation of glucosylceramide is sufficient to promote the formation and stabilization of oligomeric α-synuclein intermediates in primary cultures of human iPSC (Mazzulli et al., 2011). Knockdown of GCase in rat brain was shown to induce the accumulation of α-synuclein in the striatum (Du et al., 2015). Knockdown of GCase was also associated with decreased expression of the autophagy pathway component Beclin 1, and this effect was regulated through inactivation of protein phosphatase 2A (Du et al., 2015). These data highlight the possibility that decreases in lysosomal enzymes can initiate a feedback loop in which autophagy can be inhibited upstream of lysosomal activity. Recent work has shown that GCase activity is decreased in neurons derived from PD patients with *LRRK2* mutations (Ysselstein et al., 2019). However, an earlier study found that patients with G2019S LRRK2 mutations showed increased GCase activity in dried blood spots as compared to healthy controls or patients with other *LRRK2* mutations, suggesting that G2019S LRRK2 mutations may be associated with a distinct pathological mechanism (Alcalay et al., 2015). Pharmacological activation of GCase can stimulate the clearance of α-synuclein and restoration of lysosomal function in iPSC-derived human midbrain dopamine neurons from patients with *GBA*-related PD or idiopathic PD (Mazzulli et al., 2016). Inhibition of LRRK2 kinase activity was found to increase GCase activity in dopaminergic neurons derived from patients with either *LRRK2* or *GBA* mutations, and this increase partially reduced accumulation of α-synuclein in these neurons (Ysselstein et al., 2019). This effect was mediated through the LRRK2 substrate Rab10. Increased LRRK2 kinase activity increases Rab10 phosphorylation at Y307, inactivating Rab10 protein function and resulting in a subsequent decrease in GCase activity, an effect that could be partially reversed with a small molecule LRRK2 kinase inhibitor. It has previously been shown that α-synuclein inhibits the lysosomal activity of GCase in neurons and brain tissue of idiopathic PD patients (Mazzulli et al., 2011). These data provide convincing evidence that the lysosome provides a convergent organelle where dysfunctional LRRK2 and/or α-synuclein can have destructive effects on lysosome function, and lead to the degeneration of neurons as seen in PD. Dysfunctions in this system may also explain the susceptibility of dopaminergic neurons, as VPS35 and LRRK2 are important for vesicular trafficking and dysfunction in these proteins may lead to mistargeting of the dopamine transporter, DAT, giving rise to aberrant dopamine metabolism and oxidation (Oaks et al., 2013). Oxidized dopamine is known to reduce GCase activity (Burbulla et al., 2017), strengthening a negative feedback loop, making dopaminergic neurons uniquely susceptible to degeneration.

### Mitochondria

#### Mitochondrial Function

Mitochondria are vital organelles, which perform a myriad of functions including ATP production (Saraste, 1999), cell signaling (Weinberg et al., 2010), and control of apoptotic cell death (Peña-Blanco and García-Sáez, 2018). Dysfunctional mitochondria were first linked to PD by the observation that people who took opioid analogs contaminated with MPTP developed PD-like symptoms and showed degeneration of dopaminergic neurons in the SN (Langston et al., 1983). This was followed by post-mortem studies demonstrating that PD patients have a deficiency in complex I activity in the SN (Keeney et al., 2006) and frontal cortex (Parker et al., 2008). More recently, a number of genes encoding proteins which are responsible for autosomal recessive PD have been identified, including Parkin, PINK1, and DJ-1, all of which are involved in mitochondrial quality control by inducing clearance of dysfunctional mitochondria, a process known as mitophagy (Narendra et al., 2008, 2010; Vives-Bauza et al., 2010; McCoy and Cookson, 2012). Thus, it appears that individual subtle effects on mitochondrial function may have a cumulative effect resulting in selective neurodegeneration. There is growing evidence that LRRK2 and α-synuclein may be involved in the maintenance of normal mitochondrial function, and perturbation in either, or both, may help to explain the mitochondrial dysfunction associated with PD.

There is ample evidence to suggest that abnormal α-synuclein expression has deleterious effects on mitochondrial function. The *N*-terminus of α-synuclein has a mitochondrial inner membrane targeting sequence, where it interacts with complex I resulting in a decrease in complex I activity *in vitro* and *in vivo*, an effect which is exacerbated by overexpression of the mutant A53T form of the protein (Devi et al., 2008; Chinta et al., 2010). Aggregated α-synuclein binds directly to and inhibits the activity of translocase of the outer membrane 20 (TOM20) resulting in defective mitochondrial protein import (Di Maio et al., 2016). Other notable effects seen with overexpression in model systems include decreased mitochondrial membrane potential, decreased ATP production, and increased oxidative stress and subsequent enhanced reactive oxygen species (ROS) production (Sarafian et al., 2013; Melo et al., 2017; Grassi et al., 2018; Ludtmann et al., 2018). Accumulation of phosphorylated α-synuclein also causes defects in neuronal respiration (Wang et al., 2019). Similarly, mounting evidence suggests that LRRK2 is important for correct mitochondrial function, and around 10% of the protein localizes to the mitochondrial fraction (West et al., 2005; Biskup et al., 2006). Animal models overexpressing G2019S LRRK2 show mitochondrial abnormalities (Ramonet et al., 2011; Yue et al., 2015), and cellular models show increased ROS, increased mitochondrial fragmentation, and increased mitophagy leading to depletion of dendritic mitochondria (Niu et al., 2012; Bahnassawy et al., 2013; Cherra et al., 2013). Fibroblasts and iPSCs derived from PD patients with *LRRK2* mutations also show impairment in general mitochondrial function, including increased oxidative damage, reduced mitochondrial membrane potential, and reduced ATP production (Mortiboys et al., 2010; Sanders et al., 2014).

Despite the evidence that accumulation of α-synuclein or mutations in LRRK2 result in mitochondrial dysfunction in distinct models, the evidence that they act together is less well substantiated. Mice overexpressing α-synuclein are more susceptible to MPTP induced dopaminergic neurotoxicity (Song et al., 2004), similar to G2019S LRRK2 overexpressing mice (Karuppagounder et al., 2016) and flies, which show decreased lifespan and sensitivity in response to rotenone (another complex I inhibitor) (Ng et al., 2009). These results show that LRRK2 and α-synuclein can directly affect activity of the electron transport chain, particularly through complex I. As of yet, the evidence for them working in concert is sparse, but further investigation is warranted into how they may work together to regulate mitochondrial bioenergetics.

#### Mitochondrial Dynamics

Mitochondrial dynamics refers to the processes of mitochondrial transport, fission, and fusion. It is possible that LRRK2 and α-synuclein may interact in the disruption of this tightly controlled process, which is vital for transport of mitochondria to areas of high energy requirements such as the synapse, and maintenance of a healthy mitochondrial network (Gilad et al., 2008). Mitochondrial fission and fusion are controlled by four large GTPases which are highly conserved from yeast to mammals: mitofusins Mfn1 and Mfn2 for outer membrane fusion, Opa1 for inner membrane fusion, and Drp1 for the fission process (Otera and Mihara, 2011). Under normal physiological conditions, these proteins respond to changes in the cellular microenvironment to ensure a dynamic, interconnected mitochondrial network which meets the energy demands of the cell. However, slight perturbations in neuronal homeostasis can disrupt this machinery and lead to fragmented, punctate mitochondria or elongated reticular mitochondria. Overexpression of WT or mutant α-synuclein *in vitro* and *in vivo* alters mitochondrial fusion dynamics, resulting in rounded, fragmented mitochondria, and this effect can occur in the absence of the fission protein Drp1 (Martin et al., 2006; Nakamura et al., 2011; Xie and Chung, 2012; O’Hara et al., 2019). Indeed, the development of fragmented mitochondria, along with mitophagy marker-positive cytoplasmic inclusions containing mainly mitochondrial remnants, preceded the loss of dopaminergic neurons in an A53T α-synuclein mouse model (Chen et al., 2015). Mitochondrial fragmentation was found to require a direct interaction between α-synuclein and the outer mitochondrial membrane in iPSCs overexpressing α-synuclein (Pozo Devoto et al., 2017). Further evidence showed a specific pathogenic confirmation of α-synuclein, termed pα-syn^∗^, induces mitochondrial depolarization and fragmentation after association with mitochondria (Grassi et al., 2018).

Overexpression of LRRK2 has been shown to elicit mitochondrial fragmentation, along with increased mitochondrial localization of Drp1. These effects are enhanced with expression of mutant forms of LRRK2 and are dependent on kinase activity, but not through direct phosphorylation of Drp1 at S616 or S637, the two best studied sites involved in mediating Drp1 translocation (Wang et al., 2012). Fibroblasts from PD patients with the G2019S LRRK2 mutation also show mitochondrial fragmentation and Drp1 translocation to the mitochondria (Niu et al., 2012; Grunewald et al., 2014). Interestingly, this effect is mediated by direct phosphorylation of Drp1 by LRRK2 at a different site, T595 (Su and Qi, 2013). A more recent study identified a novel LRRK2 variant (E193K) in an Italian family, and showed that fibroblasts cultured from these patients with PD had reduced LRRK2-Drp1 binding after treatment with MPP+, and this impacted mitochondrial fission (Perez Carrion et al., 2018). It should also be noted that fibroblasts derived from a PD patient carrying the G2019S LRRK2 mutation had an elongated mitochondrial network, but the majority of studies showed the opposite effect (Mortiboys et al., 2010). Finally, another study using fibroblasts from G2019S LRRK2 PD patients and non-manifesting carriers showed that the cells from PD patients had compromised bioenergetic function and an inefficient response to bioenergetic challenge, leading to dysfunctional mitochondrial dynamics (Juarez-Flores et al., 2018). Taken together, these data suggest that LRRK2 and α-synuclein can have direct and/or indirect effects on mitochondrial dynamics. As of yet, there is little experimental evidence to explain how and if the proteins act synergistically, but it is feasible to hypothesize such a scenario. In α-synuclein-mediated PD, α-synuclein accumulation leads to complex I inhibition and subsequent increase in ROS generation (Junn and Mouradian, 2002). ROS signaling activates LRRK2, as indicated by phosphorylation of S1292, and LRRK2 then phosphorylates Rab10 at T73, inhibiting its function, leading to impaired E-L trafficking (Di Maio et al., 2018). This results in further accumulation of α-synuclein, decreased mitochondrial import by inhibition of TOM20, and decreased mitochondrial fusion as a result of the cleavage of Opa1 by Oma1 in response to decreased mitochondrial membrane potential (Zhang et al., 2014). In *LRRK2*-mediated PD, this cycle could begin with increased phosphorylation of LRRK2 substrates. Much work is needed to show if these hypothetical scenarios have any basis in disease, but there is little doubt that these proteins interfere with the core systems of mitochondrial bioenergetics and dynamics in disease states, and that disruptions in these systems may overwhelm the capacity of mitochondria to compensate for additional insults.

#### Mitophagy

Mitophagy is a type of macroautophagy that involves the selective clearance of dysfunctional mitochondria to maintain mitochondrial homeostasis. Defective mitophagy is widely associated with PD as mutations in two genes essential for the process, Parkin and PINK1, cause autosomal recessive PD and are also associated with idiopathic PD (Cookson, 2012). Some evidence supports the notion that LRRK2 and α-synuclein may also impact mitophagy. In A53T α-synuclein overexpressing mice, α-synuclein accumulates on the mitochondria and causes an increase in mitophagy and neuronal death, although this effect appears to be mediated through a Parkin-dependent pathway (Choubey et al., 2011). Miro is a mitochondrial adaptor protein, important for trafficking of mitochondria along the microtubule network but is removed from damaged mitochondria to facilitate mitochondrial clearance through mitophagy (Lee and Lu, 2014). α-Synuclein interacts with Miro via its *N*-terminus, resulting in an upregulation in Miro expression and accumulation on the mitochondria, leading to defective mitophagy. This effect can be rescued by partial reduction of Miro protein expression in human neurons and *Drosophila* (Shaltouki et al., 2018). LRRK2 has also been shown to interact with Miro in iPSC-derived neurons, where it contributed to Miro removal from mitochondria allowing mitophagy to proceed (Hsieh et al., 2016). Cells harboring the G2019S LRRK2 mutation showed delayed mitophagy initiation, while knockdown of Miro was sufficient to rescue the defects in mitophagy caused by G2019S LRRK2 (Hsieh et al., 2016). These studies open up the possibility that LRRK2 and α-synuclein interact through control of Miro expression and removal from mitochondria to alter the rate of mitophagy in damaged mitochondria (Figure 3).

### Tau

Tau is a microtubule associated protein which has a primary function in maintaining microtubule assembly but is also known to aggregate into neurofibrilliary tangles (NFT) in some neurodegenerative diseases, such as Alzheimer’s disease and PD (Gao et al., 2018). Tau contained in NFT is hyperphosphorylated and there is some evidence to suggest that LRRK2 and α-synuclein may play a role in the phosphorylation and subsequent accumulation of tau. α-Synuclein and hyperphosphorylated tau are co-localized in LB in brain tissue from PD and DLB patients and further studies have shown a direct interaction between the two proteins (Ishizawa et al., 2003; Esposito et al., 2007). Treatment of cultured cells and WT mice with MPTP results in increased phosphorylation of tau at S262, S396, and S404, but this effect was not seen in α-synuclein KO mice (Duka et al., 2006). LRRK2 may directly phosphorylate tubulin associated tau but not free tau (Kawakami et al., 2012). Studies have shown that levels of phosphorylated tau are increased in G2019S or R1441G LRRK2 transgenic mice, and tau phosphorylation is decreased in LRRK2 KO mice. Human patients with G2019S LRRK2 mutations have NFT with hyperphosphorylated tau (Gillardon, 2009; Li et al., 2009; Melrose et al., 2010; Ruffmann et al., 2012). It is unlikely that LRRK2 and α-synuclein facilitate the increased phosphorylation of tau directly in disease states, so it stands to reason that an intermediate substrate may facilitate this phosphorylation. Rab GTPases provide one such link as the G2019S mutation in LRRK2 enhances propagation of α-synuclein in *C. elegans* through increased phosphorylation of Rab35, and Rab35 plays a central role in a pathway which is essential for tau degradation (Bae et al., 2018; Vaz-Silva et al., 2018). Interestingly, G2019S LRRK2 transgenic mice also show increased neuron-to-neuron tau transmission (Nguyen et al., 2018). Another possible intermediate is GSK-3β, a kinase known to be responsible for the α-synuclein-dependent phosphorylation of tau (Duka et al., 2009). α-Synuclein binds directly to GSK-3β and forms a tripartite complex with tau *in vitro*, which initiates the phosphorylation of tau (Kawakami et al., 2011). LRRK2 also interacts with GSK-3β, and this interaction enhances the kinase activity of GSK-3β *in vitro* via a mechanism that is not dependent on LRRK2 kinase activity (Kawakami et al., 2014). G2019S LRRK2 has a higher binding affinity for GSK-3β than WT LRRK2, and it has also been shown that transgenic G2019S LRRK2 *Drosophila* neurons exhibit GSK-3β-mediated hyperphosphorylation of tau (Lin et al., 2010; Kawakami et al., 2012). The above evidence demonstrates that both LRRK2 and α-synuclein may mediate phosphorylation and subsequent aggregation of hyperphosphorylated tau, potentially enhancing the load of misfolded protein aggregates in PD brain, providing evidence as to how the two proteins may work in distinct pathways but with similar outcomes.

## Divergent Mechanisms

Apart from the above experimental evidence for direct or indirect interactions between LRRK2 and α-synuclein, there is also evidence to suggest that the two proteins do not physically or functionally interact, but instead act on distinct pathways (Figure 1D). In an interesting study, double transgenic mice expressing human A53T α-synuclein under a hindbrain selective PrP promoter were developed on either a LRRK2 KO or G2019S LRRK2 background (Daher et al., 2012). The A53T α-synuclein single transgenic mice showed some behavioral deficits which were not exacerbated with expression of G2019S LRRK2. The neuropathological phenotype that predominantly developed in the hindbrain of this A53T α-synuclein single transgenic mouse model was unaffected by the deletion of LRRK2 or overexpression of G2019S LRRK2. Since the A53T α-synuclein single transgenic mice displayed hindbrain selective α-synuclein pathology which was not affected by G2019S LRRK2 expression or LRRK2 KO, it can be concluded that LRRK2 has a non-contributory role in development of the pathological phenotype in this specific mouse model. A separate study examined the effects of LRRK2 on α-synuclein pathology in double transgenic mice co-expressing A53T α-synuclein with WT LRRK2 or G2019S LRRK2 in selected forebrain regions (high transgene expression in cortex), and in brainstem neurons under the control of the Thy-1 promoter (Herzig et al., 2012). In the initial experiments with single transgenic WT or G2019S LRRK2 mice, high LRRK2 expression levels alone failed to induce any α-synuclein pathology in mice up to 19 months of age or behavioral effects in mice up to 3–4 months of age. There were no changes in the level of phosphorylated S129 α-synuclein and the aged mice (15 months of age) showed no other signs of α-synucleinopathy. Hence, there was no link between LRRK2 overexpression and endogenous α-synuclein pathology in these mice. Subsequent experiments examining the double transgenic mice co-expressing A53T α-synuclein with WT or G2019S LRRK2 did not reveal increased levels of phosphorylated S129 α-synuclein or exacerbate the motor dysfunction observed in A53T α-synuclein single transgenic mice. These results are in contrast to the findings reported in another study in which A53T α-synuclein and LRRK2 (WT and G2019S) were co-expressed only in selected forebrain regions (high transgene expression in cortex and striatum) and showed that LRRK2 expression accelerated the progression of neuropathological abnormalities (Lin et al., 2009). In this study, unlike in the two other studies in which LRRK2 and α-synuclein were expressed in the brainstem where endogenous LRRK2 expression is low (Daher et al., 2012; Herzig et al., 2012), LRRK2 and α-synuclein were expressed in the forebrain where endogenous LRRK2 levels are high (Lin et al., 2009). Results also differed based on levels of transgene expression in cortex and striatum. Contrasting results were also reported when both genes were co-expressed only in forebrain as opposed to forebrain and brainstem. Thus, the synergistic versus separate effects of LRRK2 and α-synuclein might be dependent on the cell type and in what region of the brain they are co-expressed.

The combined effects of G2019S LRRK2 and A53T α-synuclein have also been studied in primary hippocampal and midbrain dopaminergic neurons (Henderson et al., 2018). α-Synuclein pathology was induced in primary hippocampal cultures from G2019S LRRK2 mice and non-transgenic control mice by using α-synuclein preformed fibrils (PFF). Both G2019S LRRK2 and non-transgenic primary hippocampal neurons showed similar levels of aggregated phosphorylated S129 α-synuclein 14 days post-transduction with PFF, suggesting that LRRK2 does not exacerbate α-synuclein pathology in these neurons at this timepoint. At 21 days post-transduction, mild increase in α-synuclein pathology was observed in G2019S LRRK2 neurons which was rescued by LRRK2 inhibitors. However, primary dopaminergic neurons from G2019S LRRK2 and non-transgenic mice showed no difference in levels of aggregated phosphorylated S129 α-synuclein 21 days post-transduction with PFF, indicating LRRK2 does not worsen α-synuclein pathology in dopaminergic neurons which are more relevant to PD. However, a recently published study demonstrated that the LRRK2 G2019S mutation does affect the spread of α-synuclein pathology in specific neuronal populations, supporting the observation that the synergistic versus separate effect of LRRK2 on α-synuclein pathology may depend on specific brain regions and neuronal populations (Henderson et al., 2019a).

Human genetic studies examining interactions between *SNCA* and *LRRK2* variants have not provided evidence for convergent pathways between α-synuclein and LRRK2. Specifically, potential gene-gene interactions between three PD susceptibility genes, namely *SNCA*, *LRRK2*, and *MAPT*, have been examined (Biernacka et al., 2011). One hundred and nineteen *SNCA*, *LRRK2*, and *MAPT* haplotype tagged single nucleotide polymorphisms (SNPs) and two variable number tandem repeats were genotyped in 1,098 PD patients and compared to 1,098 matched controls. Out of the 256 interaction pairs selected in this study, only 10 interaction pairs (6 *SNCA-LRRK2*, 3 *SNCA-MAPT*, and 1 *MAPT-LRRK2*) had uncorrected *p*-values of less than 0.05. However, no significant interaction was found on further statistical analysis by correcting for multiple testing and secondary analysis based on the type of control. Based on these results, no significant interaction was found between *SNCA* and *LRRK2* genes in this set of human patients. These human genetic findings taken together with the above observations using *in vitro* and *in vivo* models introduce the possibility that LRRK2 and α-synuclein act independently of each other and do not have synergistic effects on disease pathogenesis. However, the distinct pathways for LRRK2 and α-synuclein still remain to be further explored.

Currently, the most compelling evidence for distinct effects of LRRK2 and α-synuclein comes from post-mortem studies of brains from patients with LRRK2 PD. The clinical features of LRRK2 PD patients are generally indistinguishable from those with sporadic PD (Alcalay et al., 2013; Trinh et al., 2014; Marras et al., 2016). Similarly, the associated neuropathological features of these patients are often consistent with typical sporadic PD including loss of dopaminergic neurons (Schneider and Alcalay, 2017). Therefore, it was unexpected when autopsy studies revealed that an appreciable subset of LRRK2 PD cases can have dopaminergic neuron loss but lack LB pathology (Zimprich et al., 2004), suggesting that mutant LRRK2 can mediate neurodegeneration independent of large α-synuclein aggregates. Interestingly, motor features occur regardless of the presence or absence of LB in LRRK2 PD while some non-motor features, including cognitive impairment and anxiety, are associated with the presence of LB (Kalia et al., 2015), possibly indicating that LB pathology impacts the function of cortical neurons, but not dopaminergic neurons. A study of a single case of G2019S LRRK2 PD without LB revealed the presence of small soluble α-synuclein oligomers in the cortex (Gomez and Ferrer, 2010), while another study has shown very low levels of insoluble α-synuclein in 4 G2019S LRRK2 PD cases with LB compared to sporadic PD cases (Mamais et al., 2013). These results suggest that insoluble α-synuclein aggregates play a less prominent role in LRRK2 PD and smaller soluble α-synuclein oligomers may be important for neurotoxicity. Alternatively, α-synuclein may not contribute to neurodegeneration in a subset of LRRK2 PD patients. Further research is needed to determine why α-synuclein does not appear to aggregate into insoluble forms in a proportion of LRRK2 PD cases and to characterize the presence and roles of different α-synuclein aggregates in LRRK2 PD.

## Conclusion

Substantial experimental evidence points towards an interplay between LRRK2 and α-synuclein with mutant LRRK2 accelerating the progression of α-synuclein-mediated neurodegeneration. The interactions between LRRK2 and α-synuclein are indirect with much of the evidence suggesting that Rab proteins and chaperones may be mediators. Work to date also indicates that LRRK2 and α-synuclein converge on common mechanisms that lead to neuronal death, particularly by affecting the autophagy-lysosomal pathway. Current understanding of indirect and convergent mechanisms linking LRRK2 and α-synuclein has opened doors to novel therapeutic candidates that can be targeted in PD drug discovery. These include the autophagy-lysosomal pathways and mediators including, but not limited to, chaperones and Rab proteins that can be targeted to increase α-synuclein degradation and clearance. LRRK2 kinase inhibitors or LRRK2 knockdown approaches may hold promise as potential therapeutic strategies for PD as they can prevent over-phosphorylation of LRRK2 substrates, eventually aiding in restoration of the E-L system to enhance clearance of α-synuclein aggregates. Elucidating the missing components in the pathways that potentially regulate LRKK2 and α-synuclein would give a clearer idea of the actual interaction and the unknown target molecules that mediate this complex interplay. The hope is that this additional understanding would eventually open doors to new disease-modifying therapeutic interventions for PD and provide rationale for treatment stratification.

## Author Contributions

DO’H, GP, SK, and LK wrote and proofed the manuscript. SK and LK conceived of the idea for the manuscript.

## Conflict of Interest

The authors declare that the research was conducted in the absence of any commercial or financial relationships that could be construed as a potential conflict of interest.

## References

[B1] Alcalay R. N. Levy O. A. Waters C. C. Fahn S. Ford B. Kuo S. H. (2015). Glucocerebrosidase activity in Parkinson’s disease with and without GBA mutations. *Brain* 138(Pt 9), 2648–2658. 10.1093/brain/awv179 26117366 PMC4564023

[B2] Alcalay R. N. Mirelman A. Saunders-Pullman R. Tang M. X. Mejia Santana H. Raymond D. (2013). Parkinson disease phenotype in ashkenazi jews with and without LRRK2 G2019S mutations. *Mov. Disord.* 28 1966–1971. 10.1002/mds.25647 24243757 PMC3859844

[B3] Alegre-Abarrategui J. Ansorge O. Esiri M. Wade-Martins R. (2008). LRRK2 is a component of granular alpha-synuclein pathology in the brainstem of Parkinson’s disease. *Neuropathol. Appl. Neurobiol.* 34 272–283. 10.1111/j.1365-2990.2007.00888.x 17971075 PMC2833010

[B4] Alvarez-Erviti L. Rodriguez-Oroz M. C. Cooper J. M. Caballero C. Ferrer I. Obeso J. A. (2010). Chaperone-mediated autophagy markers in Parkinson disease brains. *Arch. Neurol.* 67 1464–1472. 10.1001/archneurol.2010.198 20697033

[B5] Anderson J. P. Walker D. E. Goldstein J. M. de Laat R. Banducci K. Caccavello R. J. (2006). Phosphorylation of Ser-129 is the dominant pathological modification of alpha-synuclein in familial and sporadic Lewy body disease. *J. Biol. Chem.* 281 29739–29752. 10.1074/jbc.m600933200 16847063

[B6] Angot E. Steiner J. A. Lema Tome C. M. Ekstrom P. Mattsson B. Bjorklund A. (2012). Alpha-synuclein cell-to-cell transfer and seeding in grafted dopaminergic neurons in vivo. *PLoS One* 7:e39465 10.1371/journal.pone.0039465 PMC3380846 22737239

[B7] Auluck P. K. Chan H. Y. Trojanowski J. Q. Lee V. M. Bonini N. M. (2002). Chaperone suppression of alpha-synuclein toxicity in a Drosophila model for Parkinson’s disease. *Science* 295 865–868. 10.1126/science.1067389 11823645

[B8] Bae E. J. Kim D. K. Kim C. Mante M. Adame A. Rockenstein E. (2018). LRRK2 kinase regulates alpha-synuclein propagation via RAB35 phosphorylation. *Nat. Commun.* 9:3465 10.1038/s41467-018-05958-z PMC6110743 30150626

[B9] Bahnassawy L. Nicklas S. Palm T. Menzl I. Birzele F. Gillardon F. (2013). The parkinson’s disease-associated LRRK2 mutation R1441G inhibits neuronal differentiation of neural stem cells. *Stem Cells Dev.* 22 2487–2496. 10.1089/scd.2013.0163 23600457

[B10] Ballinger C. A. Connell P. Wu Y. Hu Z. Thompson L. J. Yin L. Y. (1999). Identification of CHIP, a novel tetratricopeptide repeat-containing protein that interacts with heat shock proteins and negatively regulates chaperone functions. *Mol. Cell. Biol.* 19 4535–4545. 10.1128/mcb.19.6.4535 10330192 PMC104411

[B11] Beilina A. Rudenko I. N. Kaganovich A. Civiero L. Chau H. Kalia S. K. (2014). Unbiased screen for interactors of leucine-rich repeat kinase 2 supports a common pathway for sporadic and familial Parkinson disease. *Proc. Natl. Acad. Sci. U.S.A.* 111 2626–2631. 10.1073/pnas.1318306111 24510904 PMC3932908

[B12] Bengoa-Vergniory N. Roberts R. F. Wade-Martins R. Alegre-Abarrategui J. (2017). Alpha-synuclein oligomers: a new hope. *Acta Neuropathol.* 134 819–838. 10.1007/s00401-017-1755-1 28803412 PMC5663814

[B13] Bhuin T. Roy J. K. (2014). Rab proteins: the key regulators of intracellular vesicle transport. *Exp. Cell Res.* 328 1–19. 10.1016/j.yexcr.2014.07.027 25088255

[B14] Bieri G. Brahic M. Bousset L. Couthouis J. Kramer N. J. Ma R. (2019). LRRK2 modifies alpha-syn pathology and spread in mouse models and human neurons. *Acta Neuropathol.* 137 961–980. 10.1007/s00401-019-01995-0 30927072 PMC6531417

[B15] Biernacka J. M. Armasu S. M. Cunningham J. M. Ahlskog J. E. Chung S. J. Maraganore D. M. (2011). Do interactions between SNCA, MAPT, and LRRK2 genes contribute to Parkinson’s disease susceptibility? *Parkinsonism Relat. Disord.* 17 730–736. 10.1016/j.parkreldis.2011.07.001 21816655 PMC4723425

[B16] Biskup S. Moore D. J. Celsi F. Higashi S. West A. B. Andrabi S. A. (2006). Localization of LRRK2 to membranous and vesicular structures in mammalian brain. *Ann. Neurol.* 60 557–569. 10.1002/ana.21019 17120249

[B17] Bosgraaf L. Van Haastert P. J. (2003). Roc, a Ras/GTPase domain in complex proteins. *Biochim. Biophys. Acta* 1643 5–10. 10.1016/j.bbamcr.2003.08.008 14654223

[B18] Bourdenx M. Bezard E. Dehay B. (2014). Lysosomes and α-synuclein form a dangerous duet leading to neuronal cell death. *Front. Neuroanat.* 8:83 10.3389/fnana.2014.00083 PMC4132369 25177278

[B19] Braak H. Del Tredici K. Rüb U. de Vos R. A. Jansen Steur E. N. Braak E. (2003). Staging of brain pathology related to sporadic Parkinson’s disease. *Neurobiol. Aging* 24 197–211. 10.1016/s0197-4580(02)00065-9 12498954

[B20] Brady R. O. Kanfer J. N. Shapiro D. (1965). Metabolism of glucocerebrosides. Ii. Evidence of an enzymatic deficiency in gaucher’s disease. *Biochem. Biophys. Res. Commun.* 18 221–225. 10.1016/0006-291x(65)90743-6 14282020

[B21] Braithwaite S. P. Stock J. B. Mouradian M. M. (2012). α-Synuclein phosphorylation as a therapeutic target in Parkinson’s disease. *Rev. Neurosci.* 23 191–198. 10.1515/revneuro-2011-0067 22499677

[B22] Burbulla L. F. Song P. Mazzulli J. R. Zampese E. Wong Y. C. Jeon S. (2017). Dopamine oxidation mediates mitochondrial and lysosomal dysfunction in Parkinson’s disease. *Science* 357 1255–1261. 10.1126/science.aam9080 28882997 PMC6021018

[B23] Cao Y. L. Yang Y. P. Mao C. J. Zhang X. Q. Wang C. T. Yang J. (2017). A role of BAG3 in regulating SNCA/alpha-synuclein clearance via selective macroautophagy. *Neurobiol. Aging* 60 104–115. 10.1016/j.neurobiolaging.2017.08.023 28941726

[B24] Chartier S. Duyckaerts C. (2018). Is Lewy pathology in the human nervous system chiefly an indicator of neuronal protection or of toxicity? *Cell Tissue Res.* 373 149–160. 10.1007/s00441-018-2854-6 29869713

[B25] Chen L. Feany M. B. (2005). Alpha-synuclein phosphorylation controls neurotoxicity and inclusion formation in a Drosophila model of Parkinson disease. *Nat. Neurosci.* 8 657–663. 10.1038/nn1443 15834418

[B26] Chen L. Xie Z. Turkson S. Zhuang X. (2015). A53T human alpha-synuclein overexpression in transgenic mice induces pervasive mitochondria macroautophagy defects preceding dopamine neuron degeneration. *J. Neurosci.* 35 890–905. 10.1523/jneurosci.0089-14.2015 25609609 PMC4300331

[B27] Chen M. L. Wu R. M. (2018). LRRK 2 gene mutations in the pathophysiology of the ROCO domain and therapeutic targets for Parkinson’s disease: a review. *J. Biomed. Sci.* 25:52 10.1186/s12929-018-0454-0 PMC6000924 29903014

[B28] Cherra S. J. Steer E. III Gusdon A. M. Kiselyov K. Chu C. T. (2013). Mutant LRRK2 elicits calcium imbalance and depletion of dendritic mitochondria in neurons. *Am. J. Pathol.* 182 474–484. 10.1016/j.ajpath.2012.10.027 23231918 PMC3562730

[B29] Chinta S. J. Mallajosyula J. K. Rane A. Andersen J. K. (2010). Mitochondrial α-synuclein accumulation impairs complex I function in dopaminergic neurons and results in increased mitophagy in vivo. *Neurosci. Lett.* 486 235–239. 10.1016/j.neulet.2010.09.061 20887775 PMC2967673

[B30] Chiu C.-C. Yeh T.-H. Lai S.-C. Weng Y.-H. Huang Y.-C. Cheng Y.-C. (2016). Increased Rab35 expression is a potential biomarker and implicated in the pathogenesis of Parkinson’s disease. *Oncotarget* 7 54215–54227. 10.18632/oncotarget.11090 27509057 PMC5342336

[B31] Choubey V. Safiulina D. Vaarmann A. Cagalinec M. Wareski P. Kuum M. (2011). Mutant A53T alpha-synuclein induces neuronal death by increasing mitochondrial autophagy. *J. Biol. Chem.* 286 10814–10824. 10.1074/jbc.m110.132514 21252228 PMC3060532

[B32] Chu Y. Dodiya H. Aebischer P. Olanow C. W. Kordower J. H. (2009). Alterations in lysosomal and proteasomal markers in Parkinson’s disease: relationship to alpha-synuclein inclusions. *Neurobiol. Dis.* 35 385–398. 10.1016/j.nbd.2009.05.023 19505575

[B33] Civiero L. Cogo S. Kiekens A. Morganti C. Tessari I. Lobbestael E. (2017). PAK6 phosphorylates 14-3-3γ to regulate steady state phosphorylation of LRRK2. *Front. Mol. Neurosci.* 10:417 10.3389/fnana.2014.00417 PMC5735978 29311810

[B34] Conway K. A. Harper J. D. Lansbury P. T. (1998). Accelerated in vitro fibril formation by a mutant alpha-synuclein linked to early-onset Parkinson disease. *Nat. Med.* 4 1318–1320. 10.1038/3311 9809558

[B35] Cookson M. R. (2012). Parkinsonism due to mutations in PINK1, parkin, and DJ-1 and oxidative stress and mitochondrial pathways. *Cold Spring. Harb. Perspect. Med.* 2:a009415 10.1101/cshperspect.a009415 PMC3426824 22951446

[B36] Cookson M. R. Dauer W. Dawson T. Fon E. A. Guo M. Shen J. (2007). The roles of kinases in familial Parkinson’s disease. *J. Neurosci.* 27 11865–11868. 10.1523/jneurosci.3695-07.2007 17978026 PMC6673380

[B37] Cresto N. Gardier C. Gubinelli F. Gaillard M. C. Liot G. West A. B. (2019). The unlikely partnership between LRRK2 and α-synuclein in Parkinson’s disease. *Eur. J. Neurosci.* 49 339–363. 10.1111/ejn.14182 30269383 PMC6391223

[B38] Cuervo A. M. Stefanis L. Fredenburg R. Lansbury P. T. Sulzer D. (2004). Impaired degradation of mutant alpha-synuclein by chaperone-mediated autophagy. *Science* 305 1292–1295. 10.1126/science.1101738 15333840

[B39] Daher J. P. Abdelmotilib H. A. Hu X. Volpicelli-Daley L. A. Moehle M. S. Fraser K. B. (2015). Leucine-rich repeat kinase 2 (LRRK2) pharmacological inhibition abates alpha-synuclein gene-induced neurodegeneration. *J. Biol. Chem.* 290 19433–19444. 10.1074/jbc.m115.660001 26078453 PMC4528108

[B40] Daher J. P. Pletnikova O. Biskup S. Musso A. Gellhaar S. Galter D. (2012). Neurodegenerative phenotypes in an A53T alpha-synuclein transgenic mouse model are independent of LRRK2. *Hum. Mol. Genet.* 21 2420–2431. 10.1093/hmg/dds057 22357653 PMC3349422

[B41] Daher J. P. Volpicelli-Daley L. A. Blackburn J. P. Moehle M. S. West A. B. (2014). Abrogation of alpha-synuclein-mediated dopaminergic neurodegeneration in LRRK2-deficient rats. *Proc. Natl. Acad. Sci. U.S.A.* 111 9289–9294. 10.1073/pnas.1403215111 24927544 PMC4078806

[B42] Danzer K. M. Haasen D. Karow A. R. Moussaud S. Habeck M. Giese A. (2007). Different species of alpha-synuclein oligomers induce calcium influx and seeding. *J. Neurosci.* 27 9220–9232. 10.1523/jneurosci.2617-07.2007 17715357 PMC6672196

[B43] Dehay B. Bové J. Rodríguez-Muela N. Perier C. Recasens A. Boya P. (2010). Pathogenic lysosomal depletion in Parkinson’s disease. *J. Neurosci.* 30 12535–12544. 10.1523/jneurosci.1920-10.2010 20844148 PMC6633458

[B44] Devi L. Raghavendran V. Prabhu B. M. Avadhani N. G. Anandatheerthavarada H. K. (2008). Mitochondrial import and accumulation of alpha-synuclein impair complex I in human dopaminergic neuronal cultures and Parkinson disease brain. *J. Biol. Chem.* 283 9089–9100. 10.1074/jbc.m710012200 18245082 PMC2431021

[B45] Di Maio R. Barrett P. J. Hoffman E. K. Barrett C. W. Zharikov A. Borah A. (2016). α-Synuclein binds to TOM20 and inhibits mitochondrial protein import in Parkinson’s disease. *Sci. Transl. Med.* 8:342ra378 10.1126/scitranslmed.aaf3634 PMC5016095 27280685

[B46] Di Maio R. Hoffman E. K. Rocha E. M. Keeney M. T. Sanders L. H. De Miranda B. R. (2018). LRRK2 activation in idiopathic Parkinson’s disease. *Sci. Transl. Med.* 10:451 10.1126/scitranslmed.aar5429 PMC6344941 30045977

[B47] Dimant H. Zhu L. Kibuuka L. N. Fan Z. Hyman B. T. McLean P. J. (2014). Direct visualization of CHIP-mediated degradation of alpha-synuclein in vivo: implications for PD therapeutics. *PLoS One* 9:e92098 10.1371/journal.pone.0092098 PMC3963877 24664141

[B48] Ding X. Goldberg M. S. (2009). Regulation of LRRK2 stability by the E3 ubiquitin ligase CHIP. *PLoS One* 4:e5949 10.1371/journal.pone.005949 PMC2694275 19536328

[B49] Dinter E. Saridaki T. Nippold M. Plum S. Diederichs L. Komnig D. (2016). Rab7 induces clearance of alpha-synuclein aggregates. *J. Neurochem.* 138 758–774. 10.1111/jnc.13712 27333324

[B50] Donaldson J. G. Johnson D. L. Dutta D. (2016). Rab and Arf G proteins in endosomal trafficking and cell surface homeostasis. *Small GTPases* 7 247–251. 10.1080/21541248.2016.1212687 27416526 PMC5129904

[B51] Dougherty M. K. Morrison D. K. (2004). Unlocking the code of 14-3-3. *J. Cell Sci.* 117(Pt 10), 1875–1884. 10.1242/jcs.01171 15090593

[B52] Du T. T. Wang L. Duan C. L. Lu L. L. Zhang J. L. Gao G. (2015). GBA deficiency promotes SNCA/α-synuclein accumulation through autophagic inhibition by inactivated PPP2A. *Autophagy* 11 1803–1820. 10.1080/15548627.2015.1086055 26378614 PMC4824589

[B53] Duka T. Duka V. Joyce J. N. Sidhu A. (2009). Alpha-Synuclein contributes to GSK-3beta-catalyzed Tau phosphorylation in Parkinson’s disease models. *FASEB J.* 23 2820–2830. 10.1096/fj.08-120410 19369384 PMC2796901

[B54] Duka T. Rusnak M. Drolet R. E. Duka V. Wersinger C. Goudreau J. L. (2006). Alpha-synuclein induces hyperphosphorylation of Tau in the MPTP model of parkinsonism. *FASEB J.* 20 2302–2312. 10.1096/fj.06-6092com 17077307

[B55] Dusonchet J. Kochubey O. Stafa K. Young S. M. Zufferey R. (2011). A rat model of progressive nigral neurodegeneration induced by the Parkinson’s disease-associated G2019S mutation in LRRK2. *J. Neurosci.* 31 907–912. 10.1523/jneurosci.5092-10.2011 21248115 PMC6632940

[B56] Dzamko N. Deak M. Hentati F. Reith A. D. Prescott A. R. Alessi D. R. (2010). Inhibition of LRRK2 kinase activity leads to dephosphorylation of Ser(910)/Ser(935), disruption of 14-3-3 binding and altered cytoplasmic localization. *Biochem. J.* 430 405–413. 10.1042/bj20100784 20659021 PMC3631100

[B57] Ebrahimi-Fakhari D. Wahlster L. McLean P. J. (2011). Molecular chaperones in Parkinson’s disease–present and future. *J. Parkinsons. Dis.* 1 299–320. 10.3233/jpd-2011-11044 22279517 PMC3264060

[B58] Eguchi T. Kuwahara T. Sakurai M. Komori T. Fujimoto T. Ito G. (2018). LRRK2 and its substrate Rab GTPases are sequentially targeted onto stressed lysosomes and maintain their homeostasis. *Proc. Natl. Acad. Sci. U.S.A.* 115 E9115–E9124. 10.1073/pnas.1812196115 30209220 PMC6166828

[B59] Esposito A. Dohm C. P. Kermer P. Bahr M. Wouters F. S. (2007). alpha-Synuclein and its disease-related mutants interact differentially with the microtubule protein tau and associate with the actin cytoskeleton. *Neurobiol. Dis.* 26 521–531. 10.1016/j.nbd.2007.01.014 17408955

[B60] Fan G. H. Zhou H. Y. Yang H. Chen S. D. (2006). Heat shock proteins reduce alpha-synuclein aggregation induced by MPP+ in SK-N-SH cells. *FEBS Lett.* 580 3091–3098. 10.1016/j.febslet.2006.04.057 16678164

[B61] Friesen E. L. De Snoo M. L. Rajendran L. Kalia L. V. Kalia S. K. (2017). Chaperone-based therapies for disease modification in Parkinson’s Disease. *Parkinsons. Dis.* 2017:5015307 10.1155/2017/5015307 PMC5585656 28913005

[B62] Fujiwara H. Hasegawa M. Dohmae N. Kawashima A. Masliah E. Goldberg M. S. (2002). alpha-Synuclein is phosphorylated in synucleinopathy lesions. *Nat. Cell Biol.* 4 160–164. 10.1038/ncb748 11813001

[B63] Gan-Or Z. Amshalom I. Kilarski L. L. Bar-Shira A. Gana-Weisz M. Mirelman A. (2015). Differential effects of severe vs mild GBA mutations on Parkinson disease. *Neurology* 84 880–887. 10.1212/wnl.0000000000001315 25653295 PMC4351661

[B64] Gao Y. L. Wang N. Sun F. R. Cao X. P. Zhang W. Yu J. T. (2018). Tau in neurodegenerative disease. *Ann. Transl. Med.* 6:175 10.21037/atm.2018.04.23 PMC5994507 29951497

[B65] Gilad T. Alvaro E. Anthony J. A. M. Hibo M. Jakob D. W. Gil W. (2008). Fission and selective fusion govern mitochondrial segregation and elimination by autophagy. *EMBO J.* 27 433–446. 10.1038/sj.emboj.7601963 18200046 PMC2234339

[B66] Gillardon F. (2009). Leucine-rich repeat kinase 2 phosphorylates brain tubulin-beta isoforms and modulates microtubule stability–a point of convergence in parkinsonian neurodegeneration? *J. Neurochem.* 110 1514–1522. 10.1111/j.1471-4159.2009.06235.x 19545277

[B67] Gitler A. D. Bevis B. J. Shorter J. Strathearn K. E. Hamamichi S. Su L. J. (2008). The Parkinson’s disease protein alpha-synuclein disrupts cellular Rab homeostasis. *Proc. Natl. Acad. Sci. U.S.A.* 105 145–150. 10.1073/pnas.0710685105 18162536 PMC2224176

[B68] Goker-Alpan O. Schiffmann R. LaMarca M. E. Nussbaum R. L. McInerney-Leo A. Sidransky E. (2004). Parkinsonism among Gaucher disease carriers. *J. Med. Genet.* 41 937–940. 10.1136/jmg.2004.024455 15591280 PMC1735652

[B69] Gomez A. Ferrer I. (2010). Involvement of the cerebral cortex in Parkinson disease linked with G2019S LRRK2 mutation without cognitive impairment. *Acta Neuropathol.* 120 155–167. 10.1007/s00401-010-0669-y 20232069

[B70] Gomez-Suaga P. Rivero-Rios P. Fdez E. Blanca Ramirez M. Ferrer I. Aiastui A. (2014). LRRK2 delays degradative receptor trafficking by impeding late endosomal budding through decreasing Rab7 activity. *Hum. Mol. Genet.* 23 6779–6796. 10.1093/hmg/ddu395 25080504

[B71] Grassi D. Howard S. Zhou M. Diaz-Perez N. Urban N. T. Guerrero-Given D. (2018). Identification of a highly neurotoxic α-synuclein species inducing mitochondrial damage and mitophagy in Parkinson’s disease. *Proc. Natl. Acad. Sci. U.S.A.* 115 E2634–E2643. 10.1073/pnas.1713849115 29487216 PMC5856519

[B72] Greggio E. (2012). Role of LRRK2 kinase activity in the pathogenesis of Parkinson’s disease. *Biochem. Soc. Trans.* 40 1058–1062. 10.1042/bst20120054 22988865

[B73] Greggio E. Cookson M. R. (2009). Leucine-rich repeat kinase 2 mutations and Parkinson’s disease: three questions. *ASN Neurol.* 1:e00002 10.1042/AN20090007 PMC2695577 19570025

[B74] Greggio E. Jain S. Kingsbury A. Bandopadhyay R. Lewis P. Kaganovich A. (2006). Kinase activity is required for the toxic effects of mutant LRRK2/dardarin. *Neurobiol. Dis.* 23 329–341. 10.1016/j.nbd.2006.04.001 16750377

[B75] Grunewald A. Arns B. Meier B. Brockmann K. Tadic V. Klein C. (2014). Does uncoupling protein 2 expression qualify as marker of disease status in LRRK2-associated Parkinson’s disease? *Antioxid. Redox. Signal.* 20 1955–1960. 10.1089/ars.2013.5737 24251413 PMC3993019

[B76] Guaitoli G. Raimondi F. Gilsbach B. K. Gomez-Llorente Y. Deyaert E. Renzi F. (2016). Structural model of the dimeric Parkinson’s protein LRRK2 reveals a compact architecture involving distant interdomain contacts. *Proc. Natl. Acad. Sci. U.S.A.* 113 E4357–E4366. 10.1073/pnas.1523708113 27357661 PMC4968714

[B77] Guerreiro P. S. Huang Y. Gysbers A. Cheng D. Gai W. P. Outeiro T. F. (2013). LRRK2 interactions with alpha-synuclein in Parkinson’s disease brains and in cell models. *J. Mol. Med.* 91 513–522. 10.1007/s00109-012-0984-y 23183827 PMC3611031

[B78] Hansen C. Angot E. Bergstrom A. L. Steiner J. A. Pieri L. Paul G. (2011). alpha-Synuclein propagates from mouse brain to grafted dopaminergic neurons and seeds aggregation in cultured human cells. *J. Clin. Invest.* 121 715–725. 10.1172/jci43366 21245577 PMC3026723

[B79] Henderson M. X. Cornblath E. J. Darwich A. Zhang B. Brown H. Gathagan R. J. (2019a). Spread of alpha-synuclein pathology through the brain connectome is modulated by selective vulnerability and predicted by network analysis. *Nat. Neurosci.* 22 1248–1257. 10.1038/s41593-019-0457-5 31346295 PMC6662627

[B80] Henderson M. X. Peng C. Trojanowski J. Q. Lee V. M. Y. (2018). LRRK2 activity does not dramatically alter alpha-synuclein pathology in primary neurons. *Acta Neuropathol. Commun.* 6:45. 10.1186/s40478-018-0550-0 PMC5984465 29855356

[B81] Henderson M. X. Sengupta M. Trojanowski J. Q. Lee V. M. Y. (2019b). Alzheimer’s disease tau is a prominent pathology in LRRK2 Parkinson’s disease. *Acta Neuropathol. Commun.* 7:183 10.1186/s40478-019-0836-x PMC6858668 31733655

[B82] Henry A. G. Aghamohammadzadeh S. Samaroo H. Chen Y. Mou K. Needle E. (2015). Pathogenic LRRK2 mutations, through increased kinase activity, produce enlarged lysosomes with reduced degradative capacity and increase ATP13A2 expression. *Hum. Mol. Genet.* 24 6013–6028. 10.1093/hmg/ddv314 26251043

[B83] Herzig M. C. Bidinosti M. Schweizer T. Hafner T. Stemmelen C. Weiss A. (2012). High LRRK2 levels fail to induce or exacerbate neuronal alpha-synucleinopathy in mouse brain. *PLoS One* 7:e36581 10.1371/journal.pone.00336581 PMC3352901 22615783

[B84] Hockey L. N. Kilpatrick B. S. Eden E. R. Lin-Moshier Y. Brailoiu G. C. Brailoiu E. (2015). Dysregulation of lysosomal morphology by pathogenic LRRK2 is corrected by TPC2 inhibition. *J. Cell Sci.* 128 232–238. 10.1242/jcs.164152 25416817 PMC4294771

[B85] Hsieh C. H. Shaltouki A. Gonzalez A. E. Bettencourt da Cruz A. Burbulla L. F. St Lawrence E. (2016). Functional impairment in miro degradation and mitophagy is a shared feature in familial and sporadic Parkinson’s Disease. *Cell Stem Cell* 19 709–724. 10.1016/j.stem.2016.08.002 27618216 PMC5135570

[B86] Hutagalung A. H. Novick P. J. (2011). Role of Rab GTPases in membrane traffic and cell physiology. *Physiol. Rev.* 91 119–149. 10.1152/physrev.00059.2009 21248164 PMC3710122

[B87] Ishizawa T. Mattila P. Davies P. Wang D. Dickson D. W. (2003). Colocalization of tau and alpha-synuclein epitopes in Lewy bodies. *J. Neuropathol. Exp. Neurol.* 62 389–397. 10.1093/jnen/62.4.389 12722831

[B88] Jager S. Bucci C. Tanida I. Ueno T. Kominami E. Saftig P. (2004). Role for Rab7 in maturation of late autophagic vacuoles. *J. Cell Sci.* 117(Pt 20), 4837–4848. 10.1242/jcs.01370 15340014

[B89] Jakes R. Spillantini M. G. Goedert M. (1994). Identification of two distinct synucleins from human brain. *FEBS Lett.* 345 27–32. 10.1016/0014-5793(94)00395-5 8194594

[B90] Jeong G. R. Jang E. H. Bae J. R. Jun S. Kang H. C. Park C. H. (2018). Dysregulated phosphorylation of Rab GTPases by LRRK2 induces neurodegeneration. *Mol. Neurodegener.* 13:8 10.1186/s13024-018-0240-1 PMC5811984 29439717

[B91] Juarez-Flores D. L. Gonzalez-Casacuberta I. Ezquerra M. Bano M. Carmona-Pontaque F. Catalan-Garcia M. (2018). Exhaustion of mitochondrial and autophagic reserve may contribute to the development of LRRK2 (G2019S) -Parkinson’s disease. *J. Transl. Med.* 16:160 10.1186/s12967-018-1526-3 PMC5994110 29884186

[B92] Junn E. Mouradian M. M. (2002). Human alpha-synuclein over-expression increases intracellular reactive oxygen species levels and susceptibility to dopamine. *Neurosci. Lett.* 320 146–150. 10.1016/s0304-3940(02)00016-2 11852183

[B93] Kachergus J. Mata I. F. Hulihan M. Taylor J. P. Lincoln S. Aasly J. (2005). Identification of a novel LRRK2 mutation linked to autosomal dominant parkinsonism: evidence of a common founder across European populations. *Am. J. Hum. Genet.* 76 672–680. 10.1086/429256 15726496 PMC1199304

[B94] Kahle P. J. Neumann M. Ozmen L. Muller V. Jacobsen H. Schindzielorz A. (2000). Subcellular localization of wild-type and Parkinson’s disease-associated mutant alpha -synuclein in human and transgenic mouse brain. *J. Neurosci.* 20 6365–6373. 10.1523/jneurosci.20-17-06365.2000 10964942 PMC6772969

[B95] Kalia L. V. Kalia S. K. (2015). alpha-Synuclein and Lewy pathology in Parkinson’s disease. *Curr. Opin. Neurol.* 28 375–381. 10.1097/WCO.0000000000000215 26110807

[B96] Kalia L. V. Kalia S. K. Chau H. Lozano A. M. Hyman B. T. McLean P. J. (2011). Ubiquitinylation of α-synuclein by carboxyl terminus Hsp70-interacting protein (CHIP) is regulated by Bcl-2-associated athanogene 5 (BAG5). *PLoS One* 6:e14695 10.1371/journal.pone.0014695 PMC3040167 21358815

[B97] Kalia L. V. Kalia S. K. McLean P. J. Lozano A. M. Lang A. E. (2013). alpha-Synuclein oligomers and clinical implications for Parkinson disease. *Ann. Neurol.* 73 155–169. 10.1002/ana.23746 23225525 PMC3608838

[B98] Kalia L. V. Lang A. E. (2015). Parkinson’s disease. *Lancet* 386 896–912. 10.1016/S0140-6736(14)61393-3 25904081

[B99] Kalia L. V. Lang A. E. Hazrati L. N. Fujioka S. Wszolek Z. K. Dickson D. W. (2015). Clinical correlations with Lewy body pathology in LRRK2-related Parkinson disease. *JAMA Neurol.* 72 100–105. 10.1001/jamaneurol.2014.2704 25401511 PMC4399368

[B100] Karpinar D. P. Balija M. B. Kugler S. Opazo F. Rezaei-Ghaleh N. Wender N. (2009). Pre-fibrillar alpha-synuclein variants with impaired beta-structure increase neurotoxicity in Parkinson’s disease models. *EMBO J.* 28 3256–3268. 10.1038/emboj.2009.257 19745811 PMC2771093

[B101] Karuppagounder S. S. Xiong Y. Lee Y. Lawless M. C. Kim D. Nordquist E. (2016). LRRK2 G2019S transgenic mice display increased susceptibility to 1-methyl-4-phenyl-1,2,3,6-tetrahydropyridine (MPTP)-mediated neurotoxicity. *J. Chem. Neuroanat.* 76(Pt B), 90–97. 10.1016/j.jchemneu.2016.01.007 26808467 PMC4958044

[B102] Kawakami F. Shimada N. Ohta E. Kagiya G. Kawashima R. Maekawa T. (2014). Leucine-rich repeat kinase 2 regulates tau phosphorylation through direct activation of glycogen synthase kinase-3beta. *FEBS J.* 281 3–13. 10.1111/febs.12579 24165324

[B103] Kawakami F. Suzuki M. Shimada N. Kagiya G. Ohta E. Tamura K. (2011). Stimulatory effect of alpha-synuclein on the tau-phosphorylation by GSK-3beta. *FEBS J.* 278 4895–4904. 10.1111/j.1742-4658.2011.08389.x 21985244

[B104] Kawakami F. Yabata T. Ohta E. Maekawa T. Shimada N. Suzuki M. (2012). LRRK2 phosphorylates tubulin-associated tau but not the free molecule: LRRK2-mediated regulation of the tau-tubulin association and neurite outgrowth. *PLoS One* 7:e30834 10.1371/journal.pone.030834 PMC3267742 22303461

[B105] Kawamoto Y. Akiguchi I. Nakamura S. Honjyo Y. Shibasaki H. Budka H. (2002). 14-3-3 proteins in Lewy bodies in Parkinson disease and diffuse Lewy body disease brains. *J. Neuropathol. Exp. Neurol.* 61 245–253. 10.1093/jnen/61.3.245 11895039

[B106] Keeney P. M. Xie J. Capaldi R. A. Bennett J. P. (2006). Parkinson’s disease brain mitochondrial complex I has oxidatively damaged subunits and is functionally impaired and misassembled. *J. Neurosci.* 26 5256–5264. 10.1523/jneurosci.0984-06.2006 16687518 PMC6674236

[B107] Khan N. L. Jain S. Lynch J. M. Pavese N. Abou-Sleiman P. Holton J. L. (2005). Mutations in the gene LRRK2 encoding dardarin (PARK8) cause familial Parkinson’s disease: clinical, pathological, olfactory and functional imaging and genetic data. *Brain* 128(Pt 12), 2786–2796. 10.1093/brain/awh667 16272164

[B108] Kim S. Yun S. P. Lee S. Umanah G. E. Bandaru V. V. R. Yin X. (2018). GBA1 deficiency negatively affects physiological α-synuclein tetramers and related multimers. *Proc. Natl. Acad. Sci. U.S.A.* 115 798–803. 10.1073/pnas.1700465115 29311330 PMC5789900

[B109] Klucken J. Shin Y. Masliah E. Hyman B. T. McLean P. J. (2004). Hsp70 Reduces alpha-synuclein aggregation and toxicity. *J. Biol. Chem.* 279 25497–25502. 10.1074/jbc.m400255200 15044495

[B110] Ko H. S. Bailey R. Smith W. W. Liu Z. Shin J. H. Lee Y. I. (2009). CHIP regulates leucine-rich repeat kinase-2 ubiquitination, degradation, and toxicity. *Proc. Natl. Acad. Sci. U.S.A.* 106 2897–2902. 10.1073/pnas.0810123106 19196961 PMC2650345

[B111] Kondo K. Obitsu S. Teshima R. (2011). a-Synuclein aggregation and transmission are enhanced by leucinerich repeat kinase 2 in human neuroblastoma SH-SY5Y Cells. *Biol. Pharm. Bull.* 34 1078–1083. 10.1248/bpb.34.1078 21720016

[B112] Kordower J. H. Chu Y. Hauser R. A. Olanow C. W. Freeman T. B. (2008). Transplanted dopaminergic neurons develop PD pathologic changes: a second case report. *Mov. Disord.* 23 2303–2306. 10.1002/mds.22369 19006193

[B113] Kordower J. H. Dodiya H. B. Kordower A. M. Terpstra B. Paumier K. Madhavan L. (2011). Transfer of host-derived alpha synuclein to grafted dopaminergic neurons in rat. *Neurobiol. Dis.* 43 552–557. 10.1016/j.nbd.2011.05.001 21600984 PMC3430516

[B114] Kuwahara T. Inoue K. D’Agati V. D. Fujimoto T. Eguchi T. Saha S. (2016). LRRK2 and RAB7L1 coordinately regulate axonal morphology and lysosome integrity in diverse cellular contexts. *Sci. Rep.* 6:29945 10.1038/srep29945 PMC4947924 27424887

[B115] Langston J. Ballard P. Tetrud J. Irwin I. (1983). Chronic Parkinsonism in humans due to a product of meperidine-analog synthesis. *Science* 219 979–980. 10.1126/science.6823561 6823561

[B116] Lee H. J. Khoshaghideh F. Patel S. Lee S. J. (2004). Clearance of alpha-synuclein oligomeric intermediates via the lysosomal degradation pathway. *J. Neurosci.* 24 1888–1896. 10.1523/jneurosci.3809-03.2004 14985429 PMC6730405

[B117] Lee K. S. Lu B. (2014). The myriad roles of Miro in the nervous system: axonal transport of mitochondria and beyond. *Front. Cell Neurosci.* 8:330 10.3389/fnana.2014.0330 PMC4211407 25389385

[B118] Lee S. J. Desplats P. Lee H. J. Spencer B. Masliah E. (2012). Cell-to-cell transmission of alpha-synuclein aggregates. *Methods Mol. Biol.* 849 347–359. 10.1007/978-1-61779-551-0_23 22528101 PMC4940116

[B119] Lee S. J. Desplats P. Sigurdson C. Tsigelny I. Masliah E. (2010). Cell-to-cell transmission of non-prion protein aggregates. *Nat. Rev. Neurol.* 6 702–706. 10.1038/nrneurol.2010.145 21045796 PMC4996353

[B120] Leverenz J. B. Umar I. Wang Q. Montine T. J. McMillan P. J. Tsuang D. W. (2007). Proteomic identification of novel proteins in cortical lewy bodies. *Brain Pathol.* 17 139–145. 10.1111/j.1750-3639.2007.00048.x 17388944 PMC8095629

[B121] Li J. Y. Englund E. Holton J. L. Soulet D. Hagell P. Lees A. J. (2008). Lewy bodies in grafted neurons in subjects with Parkinson’s disease suggest host-to-graft disease propagation. *Nat. Med.* 14 501–503. 10.1038/nm1746 18391963

[B122] Li Y. Liu W. Oo T. F. Wang L. Tang Y. Jackson-Lewis V. (2009). Mutant LRRK2(R1441G) BAC transgenic mice recapitulate cardinal features of Parkinson’s disease. *Nat. Neurosci.* 12 826–828. 10.1038/nn.2349 19503083 PMC2845930

[B123] Lim S. Kim H. J. Kim D. K. Lee S. J. (2018). Non-cell-autonomous actions of alpha-synuclein: Implications in glial synucleinopathies. *Prog. Neurobiol.* 169 158–171. 10.1016/j.pneurobio.2018.06.010 30173732

[B124] Lin C. H. Tsai P. I. Wu R. M. Chien C. T. (2010). LRRK2 G2019S mutation induces dendrite degeneration through mislocalization and phosphorylation of tau by recruiting autoactivated GSK3ss. *J. Neurosci.* 30 13138–13149. 10.1523/jneurosci.1737-10.2010 20881132 PMC6633523

[B125] Lin X. Parisiadou L. Gu X. L. Wang L. Shim H. Sun L. (2009). Leucine-rich repeat kinase 2 regulates the progression of neuropathology induced by Parkinson’s-disease-related mutant alpha-synuclein. *Neuron* 64 807–827. 10.1016/j.neuron.2009.11.006 20064389 PMC2807409

[B126] Ling H. Kara E. Bandopadhyay R. Hardy J. Holton J. Xiromerisiou G. (2013). TDP-43 pathology in a patient carrying G2019S LRRK2 mutation and a novel p.*Q124E* MAPT. *Neurobiol. Aging* 34 e2885–e2889. 10.1016/j.neurobiolaging.2013.04.011 PMC3906605 23664753

[B127] Liu Z. Bryant N. Kumaran R. Beilina A. Abeliovich A. Cookson M. R. (2018). LRRK2 phosphorylates membrane-bound Rabs and is activated by GTP-bound Rab7L1 to promote recruitment to the trans-Golgi network. *Hum. Mol. Genet.* 27 385–395. 10.1093/hmg/ddx410 29177506 PMC5886198

[B128] Longo F. Mercatelli D. Novello S. Arcuri L. Brugnoli A. Vincenzi F. (2017). Age-dependent dopamine transporter dysfunction and Serine129 phospho-alpha-synuclein overload in G2019S LRRK2 mice. *Acta Neuropathol. Commun.* 5:22 10.1186/s40478-017-0426-8 PMC5351259 28292328

[B129] Ludtmann M. H. R. Angelova P. R. Horrocks M. H. Choi M. L. Rodrigues M. Baev A. Y. (2018). α-synuclein oligomers interact with ATP synthase and open the permeability transition pore in Parkinson’s disease. *Nat. Commun.* 9:2293 10.1038/s41467-018-04422-2 PMC5997668 29895861

[B130] MacLeod D. A. Rhinn H. Kuwahara T. Zolin A. Di Paolo G. McCabe B. D. (2013). RAB7L1 interacts with LRRK2 to modify intraneuronal protein sorting and Parkinson’s disease risk. *Neuron* 77 425–439. 10.1016/j.neuron.2012.11.033 23395371 PMC3646583

[B131] Mak S. K. McCormack A. L. Manning-Bog A. B. Cuervo A. M. Di Monte D. A. (2010). Lysosomal degradation of alpha-synuclein in vivo. *J. Biol. Chem.* 285 13621–13629. 10.1074/jbc.M109.074617 20200163 PMC2859524

[B132] Mamais A. Chia R. Beilina A. Hauser D. N. Hall C. Lewis P. A. (2014). Arsenite stress down-regulates phosphorylation and 14-3-3 binding of leucine-rich repeat kinase 2 (LRRK2), promoting self-association and cellular redistribution. *J. Biol. Chem.* 289 21386–21400. 10.1074/jbc.m113.528463 24942733 PMC4118103

[B133] Mamais A. Raja M. Manzoni C. Dihanich S. Lees A. Moore D. (2013). Divergent α-synuclein solubility and aggregation properties in G2019S LRRK2 Parkinson’s disease brains with Lewy Body pathology compared to idiopathic cases. *Neurobiol. Dis.* 58 183–190. 10.1016/j.nbd.2013.05.017 23747310 PMC3752970

[B134] Marchant J. S. Patel S. (2015). Two-pore channels at the intersection of endolysosomal membrane traffic. *Biochem. Soc. Trans.* 43 434–441. 10.1042/bst20140303 26009187 PMC4730950

[B135] Marras C. Alcalay R. N. Caspell-Garcia C. Coffey C. Chan P. Duda J. E. (2016). Motor and nonmotor heterogeneity of LRRK2-related and idiopathic Parkinson’s disease. *Mov. Disord.* 31 1192–1202. 10.1002/mds.26614 27091104

[B136] Martin L. J. Pan Y. Price A. C. Sterling W. Copeland N. G. Jenkins N. A. (2006). Parkinson’s disease alpha-synuclein transgenic mice develop neuronal mitochondrial degeneration and cell death. *J. Neurosci.* 26 41–50. 10.1523/jneurosci.4308-05.2006 16399671 PMC6381830

[B137] Martinez-Vicente M. Talloczy Z. Kaushik S. Massey A. C. Mazzulli J. Mosharov E. V. (2008). Dopamine-modified alpha-synuclein blocks chaperone-mediated autophagy. *J. Clin. Invest.* 118 777–788. 10.1172/JCI32806 18172548 PMC2157565

[B138] Massey A. Kiffin R. Cuervo A. M. (2004). Pathophysiology of chaperone-mediated autophagy. *Int. J. Biochem. Cell Biol.* 36 2420–2434. 10.1016/j.biocel.2004.04.010 15325582

[B139] Massey A. C. Kaushik S. Sovak G. Kiffin R. Cuervo A. M. (2006). Consequences of the selective blockage of chaperone-mediated autophagy. *Proc. Natl. Acad. Sci. U.S.A.* 103 5805–5810. 10.1073/pnas.0507436103 16585521 PMC1458654

[B140] Mazzulli J. R. Xu Y. H. Sun Y. Knight A. L. McLean P. J. Caldwell G. A. (2011). Gaucher disease glucocerebrosidase and alpha-synuclein form a bidirectional pathogenic loop in synucleinopathies. *Cell* 146 37–52. 10.1016/j.cell.2011.06.001 21700325 PMC3132082

[B141] Mazzulli J. R. Zunke F. Tsunemi T. Toker N. J. Jeon S. Burbulla L. F. (2016). Activation of β-Glucocerebrosidase reduces pathological α-synuclein and restores lysosomal function in parkinson’s patient midbrain neurons. *J. Neurosci.* 36 7693–7706. 10.1523/jneurosci.0628-16.2016 27445146 PMC4951575

[B142] McCoy M. K. Cookson M. R. (2012). Mitochondrial quality control and dynamics in Parkinson’s disease. *Antioxid. Redox. Signal.* 16 869–882. 10.1089/ars.2011.4019 21568830 PMC3292751

[B143] McFarland M. A. Ellis C. E. Markey S. P. Nussbaum R. L. (2008). Proteomics analysis identifies phosphorylation-dependent alpha-synuclein protein interactions. *Mol. Cell. Proteomics* 7 2123–2137. 10.1074/mcp.m800116-mcp200 18614564 PMC2577212

[B144] McLean P. J. Kawamata H. Shariff S. Hewett J. Sharma N. Ueda K. (2002). TorsinA and heat shock proteins act as molecular chaperones: suppression of alpha-synuclein aggregation. *J. Neurochem.* 83 846–854. 10.1046/j.1471-4159.2002.01190.x 12421356

[B145] Meade R. M. Fairlie D. P. Mason J. M. (2019). Alpha-synuclein structure and Parkinson’s disease - lessons and emerging principles. *Mol. Neurodegener.* 14:29 10.1186/s13024-019-0329-1 PMC6647174 31331359

[B146] Melo T. Q. van Zomeren K. C. Ferrari M. F. Boddeke H. W. Copray J. C. (2017). Impairment of mitochondria dynamics by human A53T alpha-synuclein and rescue by NAP (davunetide) in a cell model for Parkinson’s disease. *Exp. Brain Res.* 235 731–742. 10.1007/s00221-016-4836-9 27866262 PMC5315729

[B147] Melrose H. L. Dachsel J. C. Behrouz B. Lincoln S. J. Yue M. Hinkle K. M. (2010). Impaired dopaminergic neurotransmission and microtubule-associated protein tau alterations in human LRRK2 transgenic mice. *Neurobiol. Dis.* 40 503–517. 10.1016/j.nbd.2010.07.010 20659558 PMC2955774

[B148] Moors T. E. Hoozemans J. J. Ingrassia A. Beccari T. Parnetti L. Chartier-Harlin M. C. (2017). Therapeutic potential of autophagy-enhancing agents in Parkinson’s disease. *Mol Neurodegener.* 12:11 10.1186/s13024-017-0154-3 PMC5267440 28122627

[B149] Mortiboys H. Johansen K. K. Aasly J. O. Bandmann O. (2010). Mitochondrial impairment in patients with Parkinson disease with the G2019S mutation in LRRK2. *Neurology* 75 2017–2020. 10.1212/wnl.0b013e3181ff9685 21115957

[B150] Müller O. Sattler T. Flötenmeyer M. Schwarz H. Plattner H. Mayer A. (2000). Autophagic tubes: vacuolar invaginations involved in lateral membrane sorting and inverse vesicle budding. *J. Cell Biol.* 151 519–528. 10.1083/jcb.151.3.519 11062254 PMC2185586

[B151] Nakamura K. Nemani V. M. Azarbal F. Skibinski G. Levy J. M. Egami K. (2011). Direct membrane association drives mitochondrial fission by the Parkinson disease-associated protein alpha-synuclein. *J. Biol. Chem.* 286 20710–20726. 10.1074/jbc.m110.213538 21489994 PMC3121472

[B152] Narendra D. Tanaka A. Suen D. F. Youle R. J. (2008). Parkin is recruited selectively to impaired mitochondria and promotes their autophagy. *J. Cell Biol.* 183 795–803. 10.1083/jcb.200809125 19029340 PMC2592826

[B153] Narendra D. P. Jin S. M. Tanaka A. Suen D. F. Gautier C. A. Shen J. (2010). PINK1 is selectively stabilized on impaired mitochondria to activate Parkin. *PLoS Biol.* 8:e1000298 10.1371/journal.pone.1000298 PMC2811155 20126261

[B154] Ng C. H. Mok S. Z. Koh C. Ouyang X. Fivaz M. L. Tan E. K. (2009). Parkin protects against LRRK2 G2019S mutant-induced dopaminergic neurodegeneration in Drosophila. *J. Neurosci.* 29 11257–11262. 10.1523/jneurosci.2375-09.2009 19741132 PMC2771772

[B155] Nguyen A. P. T. Daniel G. Valdes P. Islam M. S. Schneider B. L. Moore D. J. (2018). G2019S LRRK2 enhances the neuronal transmission of tau in the mouse brain. *Hum. Mol. Genet.* 27 120–134. 10.1093/hmg/ddx389 29088368

[B156] Nichols R. J. Dzamko N. Morrice N. A. Campbell D. G. Deak M. Ordureau A. (2010). 14-3-3 binding to LRRK2 is disrupted by multiple Parkinson’s disease-associated mutations and regulates cytoplasmic localization. *Biochem. J.* 430 393–404. 10.1042/bj20100483 20642453 PMC2932554

[B157] Niu J. Yu M. Wang C. Xu Z. (2012). Leucine-rich repeat kinase 2 disturbs mitochondrial dynamics via Dynamin-like protein. *J. Neurochem.* 122 650–658. 10.1111/j.1471-4159.2012.07809.x 22639965

[B158] Oaks A. W. Marsh-Armstrong N. Jones J. M. Credle J. J. Sidhu A. (2013). Synucleins antagonize endoplasmic reticulum function to modulate dopamine transporter trafficking. *PLoS One* 8:e70872 10.1371/journal.pone.0070872 PMC3742698 23967127

[B159] O’Hara D. Davis G. M. Adlesic N. A. Hayes J. M. Davey G. P. (2019). Dichloroacetate stabilizes mitochondrial fusion dynamics in models of neurodegeneration. *Front. Mol. Neurosci.* 12:219 10.3389/fnana.2014.219 PMC6759677 31619961

[B160] Okochi M. Walter J. Koyama A. Nakajo S. Baba M. Iwatsubo T. (2000). Constitutive phosphorylation of the Parkinson’s disease associated alpha-synuclein. *J. Biol. Chem.* 275 390–397. 10.1074/jbc.275.1.390 10617630

[B161] Orenstein S. J. Kuo S. H. Tasset I. Arias E. Koga H. Fernandez-Carasa I. (2013). Interplay of LRRK2 with chaperone-mediated autophagy. *Nat. Neurosci.* 16 394–406. 10.1038/nn.3350 23455607 PMC3609872

[B162] Osellame L. D. Duchen M. R. (2014). Quality control gone wrong: mitochondria, lysosomal storage disorders and neurodegeneration. *Br. J. Pharmacol.* 171 1958–1972. 10.1111/bph.12453 24116849 PMC3976615

[B163] Ostrerova N. Petrucelli L. Farrer M. Mehta N. Choi P. Hardy J. (1999). alpha-Synuclein shares physical and functional homology with 14-3-3 proteins. *J. Neurosci.* 19 5782–5791. 10.1523/jneurosci.19-14-05782.1999 10407019 PMC6783081

[B164] Otera H. Mihara K. (2011). Molecular mechanisms and physiologic functions of mitochondrial dynamics. *J. Biochem.* 149 241–251. 10.1093/jb/mvr002 21233142

[B165] Paisan-Ruiz C. Jain S. Evans E. W. Gilks W. P. Simon J. van der Brug M. (2004). Cloning of the gene containing mutations that cause PARK8-linked Parkinson’s disease. *Neuron* 44 595–600. 10.1016/j.neuron.2004.10.023 15541308

[B166] Parker W. D. Parks J. K. Swerdlow R. H. (2008). Complex I deficiency in Parkinson’s disease frontal cortex. *Brain Res.* 1189 215–218. 10.1016/j.brainres.2007.10.061 18061150 PMC2295283

[B167] Peña-Blanco A. García-Sáez A. J. (2018). Bax, Bak and beyond - mitochondrial performance in apoptosis. *FEBS J.* 285 416–431. 10.1111/febs.14186 28755482

[B168] Perera R. M. Zoncu R. (2016). The Lysosome as a Regulatory Hub. *Annu. Rev. Cell Dev. Biol.* 32 223–253. 10.1146/annurev-cellbio-111315-125125 27501449 PMC9345128

[B169] Perez Carrion M. Pischedda F. Biosa A. Russo I. Straniero L. Civiero L. (2018). The LRRK2 variant E193K prevents mitochondrial fission upon MPP+ treatment by altering LRRK2 binding to DRP1. *Front. Mol. Neurosci.* 11:64 10.3389/fnana.2014.064 PMC5835904 29541021

[B170] Periquet M. Fulga T. Myllykangas L. Schlossmacher M. G. Feany M. B. (2007). Aggregated alpha-synuclein mediates dopaminergic neurotoxicity in vivo. *J. Neurosci.* 27 3338–3346. 10.1523/jneurosci.0285-07.2007 17376994 PMC6672466

[B171] Perry G. Zhu X. Babar A. K. Siedlak S. L. Yang Q. Ito G. (2008). Leucine-rich repeat kinase 2 colocalizes with alpha-synuclein in Parkinson’s disease, but not tau-containing deposits in tauopathies. *Neurodegener. Dis.* 5 222–224. 10.1159/000113708 18322396 PMC2677749

[B172] Pfeffer S. R. (2018). LRRK2 and Rab GTPases. *Biochem. Soc. Trans.* 46 1707–1712. 10.1042/bst20180470 30467121

[B173] Pineda A. Burré J. (2017). Modulating membrane binding of α-synuclein as a therapeutic strategy. *Proc. Natl. Acad. Sci. U.S.A.* 114 1223–1225. 10.1073/pnas.1620159114 28126719 PMC5307480

[B174] Platt F. M. (2014). Sphingolipid lysosomal storage disorders. *Nature* 510 68–75. 10.1038/nature13476 24899306

[B175] Polymeropoulos M. H. Lavedan C. Leroy E. Ide S. E. (1997). Mutation in the α-synuclein gene identified in families with Parkinson’s disease. *Science* 76 2045–2047. 10.1126/science.276.5321.2045 9197268

[B176] Pozo Devoto V. M. Dimopoulos N. Alloatti M. Pardi M. B. Saez T. M. Otero M. G. (2017). αSynuclein control of mitochondrial homeostasis in human-derived neurons is disrupted by mutations associated with Parkinson’s disease. *Sci. Rep.* 7:5042 10.1038/s41598-017-05334-9 PMC5506004 28698628

[B177] Qing H. Wong W. McGeer E. G. McGeer P. L. (2009). Lrrk2 phosphorylates alpha synuclein at serine 129: Parkinson disease implications. *Biochem. Biophys. Res. Commun.* 387 149–152. 10.1016/j.bbrc.2009.06.142 19576176

[B178] Ramonet D. Daher J. P. Lin B. M. Stafa K. Kim J. Banerjee R. (2011). Dopaminergic neuronal loss, reduced neurite complexity and autophagic abnormalities in transgenic mice expressing G2019S mutant LRRK2. *PLoS One* 6:e18568 10.1371/journal.pone.0018568 PMC3071839 21494637

[B179] Ravikumar B. Futter M. Jahreiss L. Korolchuk V. I. Lichtenberg M. Luo S. (2009). Mammalian macroautophagy at a glance. *J. Cell Sci.* 122(Pt 11), 1707–1711. 10.1242/jcs.031773 19461070 PMC2684830

[B180] Reyes J. F. Olsson T. T. Lamberts J. T. Devine M. J. Kunath T. Brundin P. (2015). A cell culture model for monitoring α-synuclein cell-to-cell transfer. *Neurobiol. Dis.* 77 266–275. 10.1016/j.nbd.2014.07.003 25046995

[B181] Rudenko I. N. Kaganovich A. Langston R. G. Beilina A. Ndukwe K. Kumaran R. (2017). The G2385R risk factor for Parkinson’s disease enhances CHIP-dependent intracellular degradation of LRRK2. *Biochem. J.* 474 1547–1558. 10.1042/bcj20160909 28320779 PMC6178381

[B182] Ruffmann C. Giaccone G. Canesi M. Bramerio M. Goldwurm S. Gambacorta M. (2012). Atypical tauopathy in a patient with LRRK2-G2019S mutation and tremor-dominant Parkinsonism. *Neuropathol. Appl. Neurobiol.* 38 382–386. 10.1111/j.1365-2990.2011.01216.x 21883375

[B183] Sanders L. H. Laganiere J. Cooper O. Mak S. K. Vu B. J. Huang Y. A. (2014). LRRK2 mutations cause mitochondrial DNA damage in iPSC-derived neural cells from Parkinson’s disease patients: reversal by gene correction. *Neurobiol. Dis.* 62 381–386. 10.1016/j.nbd.2013.10.013 24148854 PMC3877733

[B184] Sarafian T. A. Ryan C. M. Souda P. Masliah E. Kar U. K. Vinters H. V. (2013). Impairment of mitochondria in adult mouse brain overexpressing predominantly full-length, N-terminally acetylated human alpha-synuclein. *PLoS One* 8:e63557 10.1371/journal.pone.0063557 PMC3646806 23667637

[B185] Saraste M. (1999). Oxidative phosphorylation at the fin de siecle. *Science* 283 1488–1493. 10.1126/science.283.5407.1488 10066163

[B186] Schapansky J. Khasnavis S. DeAndrade M. P. Nardozzi J. D. Falkson S. R. Boyd J. D. (2018). Familial knockin mutation of LRRK2 causes lysosomal dysfunction and accumulation of endogenous insoluble α-synuclein in neurons. *Neurobiol. Dis.* 111 26–35. 10.1016/j.nbd.2017.12.005 29246723 PMC5803451

[B187] Schneider S. A. Alcalay R. N. (2017). Neuropathology of genetic synucleinopathies with parkinsonism: Review of the literature. *Mov. Disord.* 32 1504–1523. 10.1002/mds.27193 29124790 PMC5726430

[B188] Seol W. Nam D. Son I. (2019). Rab GTPases as Physiological Substrates of LRRK2 Kinase. *Exp. Neurobiol.* 28 134–145. 10.5607/en.2019.28.2.134 31138985 PMC6526114

[B189] Shahmoradian S. H. Lewis A. J. Genoud C. Hench J. Moors T. E. Navarro P. P. (2019). Lewy pathology in Parkinson’s disease consists of crowded organelles and lipid membranes. *Nat. Neurosci.* 22 1099–1109. 10.1038/s41593-019-0423-2 31235907

[B190] Shaltouki A. Hsieh C. H. Kim M. J. Wang X. (2018). Alpha-synuclein delays mitophagy and targeting Miro rescues neuron loss in Parkinson’s models. *Acta Neuropathol.* 136 607–620. 10.1007/s00401-018-1873-4 29923074 PMC6123262

[B191] Sharon R. Bar-Joseph I. Frosch M. P. Walsh D. M. Hamilton J. A. Selkoe D. J. (2003). The formation of highly soluble oligomers of alpha-synuclein is regulated by fatty acids and enhanced in Parkinson’s disease. *Neuron* 37 583–595. 10.1016/s0896-6273(03)00024-2 12597857

[B192] Sheng Z. Zhang S. Bustos D. Kleinheinz T. Le Pichon C. E. Dominguez S. L. (2012). Ser1292 autophosphorylation is an indicator of LRRK2 kinase activity and contributes to the cellular effects of PD mutations. *Sci. Transl. Med.* 4:164ra161 10.1126/scitranslmed.3004485 23241745

[B193] Shih A. Y. Johnson D. A. Wong G. Kraft A. D. Jiang L. Erb H. (2003). Coordinate regulation of glutathione biosynthesis and release by Nrf2-expressing glia potently protects neurons from oxidative stress. *J. Neurosci.* 23 3394–3406. 10.1523/jneurosci.23-08-03394.2003 12716947 PMC6742304

[B194] Shin Y. Klucken J. Patterson C. Hyman B. T. McLean P. J. (2005). The co-chaperone carboxyl terminus of Hsp70-interacting protein (CHIP) mediates alpha-synuclein degradation decisions between proteasomal and lysosomal pathways. *J. Biol. Chem.* 280 23727–23734. 10.1074/jbc.m503326200 15845543

[B195] Sidransky E. Nalls M. A. Aasly J. O. Aharon-Peretz J. Annesi G. Barbosa E. R. (2009). Multicenter analysis of glucocerebrosidase mutations in Parkinson’s disease. *N. Engl. J. Med.* 361 1651–1661. 10.1056/NEJMoa0901281 19846850 PMC2856322

[B196] Simón-Sánchez J. Schulte C. Bras J. M. Sharma M. Gibbs J. R. Berg D. (2009). Genome-wide association study reveals genetic risk underlying Parkinson’s disease. *Nat. Genet.* 41 1308–1312. 10.1038/ng.487 19915575 PMC2787725

[B197] Singleton A. B. (2005). Altered alpha-synuclein homeostasis causing Parkinson’s disease: the potential roles of dardarin. *Trends Neurosci.* 28 416–421. 10.1016/j.tins.2005.05.009 15955578

[B198] Skibinski G. Hwang V. Ando D. M. Daub A. Lee A. K. Ravisankar A. (2017). Nrf2 mitigates LRRK2- and alpha-synuclein-induced neurodegeneration by modulating proteostasis. *Proc. Natl. Acad. Sci. U.S.A.* 114 1165–1170. 10.1073/pnas.1522872114 28028237 PMC5293055

[B199] Smith W. W. Pei Z. Jiang H. Dawson V. L. Dawson T. M. Ross C. A. (2006). Kinase activity of mutant LRRK2 mediates neuronal toxicity. *Nat. Neurosci.* 9 1231–1233. 10.1038/nn1776 16980962

[B200] Song D. D. Shults C. W. Sisk A. Rockenstein E. Masliah E. (2004). Enhanced substantia nigra mitochondrial pathology in human alpha-synuclein transgenic mice after treatment with MPTP. *Exp. Neurol.* 186 158–172. 10.1016/s0014-4886(03)00342-x 15026254

[B201] Song J. L. Testa M. (2018). “The function of Rab35 in development and disease,” in *Peripheral Membrane Proteins*, ed. Tanabe S. (London: Intechopen). 10.5772/intechopen.75168

[B202] Spillantini M. G. Schmidt M. L. Lee V. M. Trojanowski J. Q. Jakes R. Goedert M. (1997). Alpha-synuclein in Lewy bodies. *Nature* 388 839–840. 10.1038/42166 9278044

[B203] Steger M. Diez F. Dhekne H. S. Lis P. Nirujogi R. S. Karayel O. (2017). Systematic proteomic analysis of LRRK2-mediated Rab GTPase phosphorylation establishes a connection to ciliogenesis. *eLife* 6:e31012 10.7554/eLife.31012 PMC5695910 29125462

[B204] Steger M. Tonelli F. Ito G. Davies P. Trost M. Vetter M. (2016). Phosphoproteomics reveals that Parkinson’s disease kinase LRRK2 regulates a subset of Rab GTPases. *eLife* 5:e12813 10.7554/eLife.12813 PMC4769169 26824392

[B205] Stirnemann J. Belmatoug N. Camou F. Serratrice C. Froissart R. Caillaud C. (2017). A review of gaucher disease pathophysiology, clinical presentation and treatments. *Int. J. Mol. Sci.* 18:E441 10.3390/ijms18020441 PMC5343975 28218669

[B206] Su Y. C. Qi X. (2013). Inhibition of excessive mitochondrial fission reduced aberrant autophagy and neuronal damage caused by LRRK2 G2019S mutation. *Hum. Mol. Genet.* 22 4545–4561. 10.1093/hmg/ddt301 23813973

[B207] Suresh S. N. Chavalmane A. K. Dj V. Yarreiphang H. Rai S. Paul A. (2017). A novel autophagy modulator 6-Bio ameliorates SNCA/alpha-synuclein toxicity. *Autophagy* 13 1221–1234. 10.1080/15548627.2017.1302045 28350199 PMC5529071

[B208] Suresh S. N. Chavalmane A. K. Pillai M. Ammanathan V. Vidyadhara D. J. Yarreiphang H. (2018). Modulation of autophagy by a small molecule inverse agonist of erralpha is neuroprotective. *Front. Mol. Neurosci.* 11:109 10.3389/fnana.2014.00109 PMC5900053 29686608

[B209] Taguchi Y. V. Liu J. Ruan J. Pacheco J. Zhang X. Abbasi J. (2017). Glucosylsphingosine promotes α-Synuclein pathology in mutant GBA-associated parkinson’s disease. *J. Neurosci.* 37 9617–9631. 10.1523/jneurosci.1525-17.2017 28847804 PMC5628407

[B210] Tang B. L. (2017). Rabs, Membrane Dynamics, and Parkinson’s Disease. *J. Cell. Physiol.* 232 1626–1633. 10.1002/jcp.25713 27925204

[B211] Tang F. L. Erion J. R. Tian Y. Liu W. Yin D. M. Ye J. (2015). VPS35 in dopamine neurons is required for endosome-to-Golgi retrieval of Lamp2a, a receptor of chaperone-mediated autophagy that is critical for α-Synuclein degradation and prevention of pathogenesis of Parkinson’s Disease. *J. Neurosci.* 35 10613–10628. 10.1523/JNEUROSCI.0042-15.2015 26203154 PMC4510296

[B212] Tayebi N. Callahan M. Madike V. Stubblefield B. K. Orvisky E. Krasnewich D. (2001). Gaucher disease and parkinsonism: a phenotypic and genotypic characterization. *Mol. Genet. Metab.* 73 313–321. 10.1006/mgme.2001.3201 11509013

[B213] Tenreiro S. Eckermann K. Outeiro T. F. (2014). Protein phosphorylation in neurodegeneration: friend or foe? *Front. Mol. Neurosci.* 7:42 10.3389/fnana.2014.00042 PMC4026737 24860424

[B214] Tofaris G. K. Razzaq A. Ghetti B. Lilley K. S. Spillantini M. G. (2003). Ubiquitination of alpha-synuclein in Lewy bodies is a pathological event not associated with impairment of proteasome function. *J. Biol. Chem.* 278 44405–44411. 10.1074/jbc.M308041200 12923179

[B215] Tong Y. Yamaguchi H. Giaime E. Boyle S. Kopan R. Kelleher R. J. (2010). Loss of leucine-rich repeat kinase 2 causes impairment of protein degradation pathways, accumulation of alpha-synuclein, and apoptotic cell death in aged mice. *Proc. Natl. Acad. Sci. U.S.A.* 107 9879–9884. 10.1073/pnas.1004676107 20457918 PMC2906862

[B216] Trinh J. Amouri R. Duda J. E. Morley J. F. Read M. Donald A. (2014). Comparative study of Parkinson’s disease and leucine-rich repeat kinase 2 p.*G2019S* parkinsonism. *Neurobiol. Aging* 35 1125–1131. 10.1016/j.neurobiolaging.2013.11.015 24355527

[B217] Tsika E. Nguyen A. P. Dusonchet J. Colin P. Schneider B. L. Moore D. J. (2015). Adenoviral-mediated expression of G2019S LRRK2 induces striatal pathology in a kinase-dependent manner in a rat model of Parkinson’s disease. *Neurobiol. Dis.* 77 49–61. 10.1016/j.nbd.2015.02.019 25731749

[B218] Vaz-Silva J. Gomes P. Jin Q. Zhu M. Zhuravleva V. Quintremil S. (2018). Endolysosomal degradation of Tau and its role in glucocorticoid-driven hippocampal malfunction. *EMBO J.* 37:e99084 10.15252/embj.201899084 PMC6187216 30166454

[B219] Vives-Bauza C. Zhou C. Huang Y. Cui M. de Vries R. L. Kim J. (2010). PINK1-dependent recruitment of Parkin to mitochondria in mitophagy. *Proc. Natl. Acad. Sci. U.S.A.* 107 378–383. 10.1073/pnas.0911187107 19966284 PMC2806779

[B220] Vogiatzi T. Xilouri M. Vekrellis K. Stefanis L. (2008). Wild type alpha-synuclein is degraded by chaperone-mediated autophagy and macroautophagy in neuronal cells. *J. Biol. Chem.* 283 23542–23556. 10.1074/jbc.M801992200 18566453 PMC2527094

[B221] Volpicelli-Daley L. A. Abdelmotilib H. Liu Z. Stoyka L. Daher J. P. Milnerwood A. J. (2016). G2019S-LRRK2 expression augments alpha-synuclein sequestration into inclusions in neurons. *J. Neurosci.* 36 7415–7427. 10.1523/JNEUROSCI.3642-15.2016 27413152 PMC4945663

[B222] Volpicelli-Daley L. A. Luk K. C. Patel T. P. Tanik S. A. Riddle D. M. Stieber A. (2011). Exogenous alpha-synuclein fibrils induce Lewy body pathology leading to synaptic dysfunction and neuron death. *Neuron* 72 57–71. 10.1016/j.neuron.2011.08.033 21982369 PMC3204802

[B223] Walker D. G. Lue L. F. Adler C. H. Shill H. A. Caviness J. N. Sabbagh M. N. (2013). Changes in properties of serine 129 phosphorylated alpha-synuclein with progression of Lewy-type histopathology in human brains. *Exp. Neurol.* 240 190–204. 10.1016/j.expneurol.2012.11.020 23201181 PMC3720241

[B224] Wang B. Underwood R. Kamath A. Britain C. McFerrin M. B. McLean P. J. (2018). 14-3-3 proteins reduce cell-to-cell transfer and propagation of pathogenic alpha-synuclein. *J. Neurosci.* 38 8211–8232. 10.1523/JNEUROSCI.1134-18.2018 30093536 PMC6146494

[B225] Wang X. Becker K. Levine N. Zhang M. Lieberman A. P. Moore D. J. (2019). Pathogenic alpha-synuclein aggregates preferentially bind to mitochondria and affect cellular respiration. *Acta Neuropathol. Commun.* 7:41 10.1186/s40478-019-0696-4 PMC6419482 30871620

[B226] Wang X. Yan M. H. Fujioka H. Liu J. Wilson-Delfosse A. Chen S. G. (2012). LRRK2 regulates mitochondrial dynamics and function through direct interaction with DLP1. *Hum. Mol. Genet.* 21 1931–1944. 10.1093/hmg/dds003 22228096 PMC3315202

[B227] Waxman E. A. Giasson B. I. (2011). Characterization of kinases involved in the phosphorylation of aggregated alpha-synuclein. *J. Neurosci. Res.* 89 231–247. 10.1002/jnr.22537 21162130 PMC4484797

[B228] Webb J. L. Ravikumar B. Atkins J. Skepper J. N. Rubinsztein D. C. (2003). Alpha-Synuclein is degraded by both autophagy and the proteasome. *J. Biol. Chem.* 278 25009–25013. 10.1074/jbc.M300227200 12719433

[B229] Weinberg F. Hamanaka R. Wheaton W. W. Weinberg S. Joseph J. Lopez M. (2010). Mitochondrial metabolism and ROS generation are essential for Kras-mediated tumorigenicity. *Proc. Natl. Acad. Sci. U.S.A.* 107 8788–8793. 10.1073/pnas.1003428107 20421486 PMC2889315

[B230] West A. B. Moore D. J. Biskup S. Bugayenko A. Smith W. W. Ross C. A. (2005). Parkinson’s disease-associated mutations in leucine-rich repeat kinase 2 augment kinase activity. *Proc. Natl. Acad. Sci. U.S.A.* 102 16842–16847. 10.1073/pnas.0507360102 16269541 PMC1283829

[B231] West A. B. Moore D. J. Choi C. Andrabi S. A. Li X. Dikeman D. (2007). Parkinson’s disease-associated mutations in LRRK2 link enhanced GTP-binding and kinase activities to neuronal toxicity. *Hum. Mol. Genet.* 16 223–232. 10.1093/hmg/ddl471 17200152

[B232] Winner B. Jappelli R. Maji S. K. Desplats P. A. Boyer L. Aigner S. (2011). In vivo demonstration that α-synuclein oligomers are toxic. *Proc. Natl. Acad. Sci. U.S.A.* 108 4194–4199. 10.1073/pnas.1100976108 21325059 PMC3053976

[B233] Winslow A. R. Chen C. W. Corrochano S. Acevedo-Arozena A. Gordon D. E. Peden A. A. (2010). α-Synuclein impairs macroautophagy: implications for Parkinson’s disease. *J. Cell Biol.* 190 1023–1037. 10.1083/jcb.201003122 20855506 PMC3101586

[B234] Wszolek Z. K. Pfeiffer R. F. Tsuboi Y. Uitti R. J. McComb R. D. Stoessl A. J. (2004). Autosomal dominant parkinsonism associated with variable synuclein and tau pathology. *Neurology* 62 1619–1622. 10.1212/01.wnl.0000125015.06989.db 15136696

[B235] Xie W. Chung K. K. (2012). Alpha-synuclein impairs normal dynamics of mitochondria in cell and animal models of Parkinson’s disease. *J. Neurochem.* 122 404–414. 10.1111/j.1471-4159.2012.07769.x 22537068

[B236] Xiong Y. Neifert S. Karuppagounder S. S. Stankowski J. N. Lee B. D. Grima J. C. (2017). Overexpression of Parkinson’s Disease-associated mutation LRRK2 G2019S in mouse forebrain induces behavioral deficits and alpha-synuclein pathology. *eNeuro* 4:ENEURO.0004-17.2017 10.1523/ENEURO.0004-17.2017 PMC5355896 28321439

[B237] Xu J. Kao S. Y. Lee F. J. Song W. Jin L. W. Yankner B. A. (2002). Dopamine-dependent neurotoxicity of alpha-synuclein: a mechanism for selective neurodegeneration in Parkinson disease. *Nat. Med.* 8 600–606. 10.1038/nm0602-600 12042811

[B238] Xu Z. Graham K. Foote M. Liang F. Rizkallah R. Hurt M. (2013). 14-3-3 protein targets misfolded chaperone-associated proteins to aggresomes. *J. Cell Sci.* 126(Pt 18), 4173–4186. 10.1242/jcs.126102 23843611 PMC3772389

[B239] Yacoubian T. A. Slone S. R. Harrington A. J. Hamamichi S. Schieltz J. M. Caldwell K. A. (2010). Differential neuroprotective effects of 14-3-3 proteins in models of Parkinson’s disease. *Cell Death Dis.* 1:e2 10.1038/cddis.2009.4 PMC2998343 21152247

[B240] Ysselstein D. Nguyen M. Young T. J. Severino A. Schwake M. Merchant K. (2019). LRRK2 kinase activity regulates lysosomal glucocerebrosidase in neurons derived from Parkinson’s disease patients. *Nat. Commun.* 10:5570 10.1038/s41467-019-13413-w PMC6895201 31804465

[B241] Yue M. Hinkle K. M. Davies P. Trushina E. Fiesel F. C. Christenson T. A. (2015). Progressive dopaminergic alterations and mitochondrial abnormalities in LRRK2 G2019S knock-in mice. *Neurobiol. Dis.* 78 172–195. 10.1016/j.nbd.2015.02.031 25836420 PMC4526103

[B242] Zhang K. Li H. Song Z. (2014). Membrane depolarization activates the mitochondrial protease OMA1 by stimulating self-cleavage. *EMBO Rep.* 15 576–585. 10.1002/embr.201338240 24719224 PMC4210089

[B243] Zhen Y. Stenmark H. (2015). Cellular functions of Rab GTPases at a glance. *J. Cell Sci.* 128 3171–3176. 10.1242/jcs.166074 26272922

[B244] Zheng X. Y. Yang M. Tan J. Q. Pan Q. Long Z. G. Dai H. P. (2008). Screening of LRRK2 interactants by yeast 2-hybrid analysis. *Zhong Nan Da Xue Xue Bao Yi Xue Ban* 33 883–891. 19001729

[B245] Zimprich A. Biskup S. Leitner P. Lichtner P. Farrer M. Lincoln S. (2004). Mutations in LRRK2 cause autosomal-dominant parkinsonism with pleomorphic pathology. *Neuron* 44 601–607. 10.1016/j.neuron.2004.11.005 15541309

